# Ca^2+^ Release by IP_3_ Receptors Is Required to Orient the Mitotic Spindle

**DOI:** 10.1016/j.celrep.2020.108483

**Published:** 2020-12-15

**Authors:** Raul Lagos-Cabré, Adelina Ivanova, Colin W. Taylor

**Affiliations:** 1 Department of Pharmacology, University of Cambridge, Tennis Court Road, Cambridge CB2 1PD, UK

**Keywords:** Ca^2+^ signal, centrosome, endoplasmic reticulum, IP_3_ receptor, metaphase, microtubule, mitosis, NuMA, spindle

## Abstract

The mitotic spindle distributes chromosomes evenly to daughter cells during mitosis. The orientation of the spindle, guided by internal and external cues, determines the axis of cell division and thereby contributes to tissue morphogenesis. Progression through mitosis requires local Ca^2+^ signals at critical steps, and because store-operated Ca^2+^ entry is inhibited during mitosis, those signals probably require Ca^2+^ release through inositol 1,4,5-trisphosphate receptors (IP_3_Rs). In cells without IP_3_Rs, astral microtubules around the daughter centrosome are shorter than those at the mother centrosome, and the mitotic spindle fails to align with the substratum during metaphase. The misalignment is due to the spindle ineffectively detecting internal cues rather than a failure of cells to recognize the substratum. Expression of type 3 IP_3_R is sufficient to rescue spindle alignment, but only if the IP_3_R has a functional pore. We conclude that Ca^2+^ signals evoked by IP_3_Rs are required to orient the mitotic spindle.

## Highlights

• IP_3_ receptors are required for mitotic spindle orientation
• Only IP_3_ receptors with a functional channel restore spindle orientation
• Ca^2+^ release through IP_3_ receptors is required for spindle orientation

## Graphical Abstract

**Figure:**
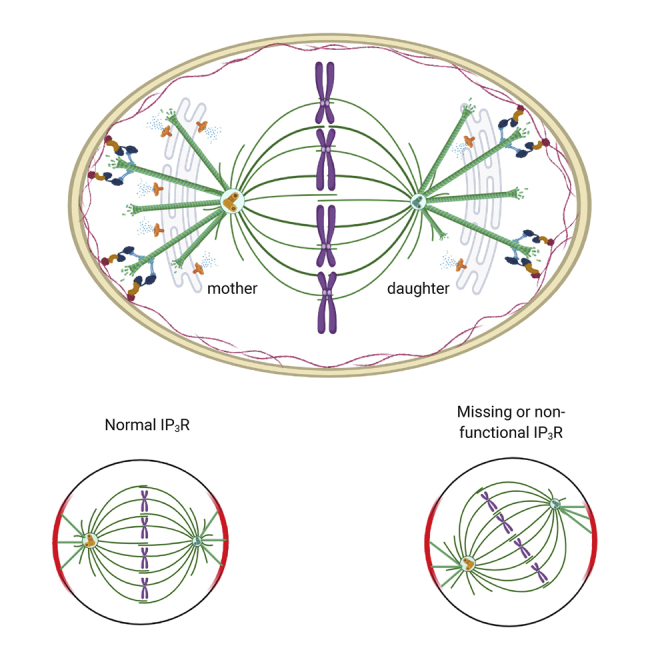

## Teaser

Lagos-Cabré et al. show that Ca^2+^ release by IP_3_ receptors (IP_3_Rs) regulates spindle orientation during metaphase. Spindles misalign in cells without IP_3_Rs. Alignment is restored by expression of IP_3_Rs but not by IP_3_Rs that cannot release Ca^2+^. Ca^2+^ release by IP_3_Rs regulates astral microtubules and thereby spindle alignment.

## Introduction

During mitosis, centrosomes nucleate microtubules to form the mitotic spindle, which then distributes chromatids equally to daughter cells. The orientation of the spindle is important because it determines the plane of cell division and which of the daughter cells will receive the oldest (mother) centrosome (Bergstralh et al., 2017; di Pietro et al., 2016). Some stem cells, for example, selectively inherit the mother centrosome (Pelletier and Yamashita, 2012). For symmetric cell divisions, spindle alignment ensures that cellular components are shared equally between daughter cells, whereas for asymmetric divisions, spindle orientation determines whether one or both cells remain attached to the basement membrane (Hehnly et al., 2015; Lagos-Cabré and Moreno, 2008). Hence, spindle orientation ensures effective mitosis; it determines cell fate and which cells remain stem cells (Pelletier and Yamashita, 2012). Aberrant spindle alignment is associated with defective morphogenesis (Hehnly et al., 2015; Seldin et al., 2016), delamination of epithelia (Nakajima et al., 2013), and cancer (Bergstralh et al., 2017). Spindle positioning is achieved by interaction of astral microtubules emanating from the centrosomes with a protein complex anchored to the cell cortex. In vertebrates, this ternary complex includes nuclear mitotic apparatus (NuMA), LGN (for leucine-glycine-asparagine motifs), and the G-protein Gα_i_ (Bergstralh et al., 2017). How cells regulate the interactions of astral microtubules with the cell cortex to ensure effective orientation of the mitotic spindle is not fully understood (di Pietro et al., 2016).

Ca^2+^ signals are associated with many key steps during mitosis, notably, at nuclear envelope breakdown, the transition from metaphase to anaphase and during cytokinesis (Humeau et al., 2018; Poenie et al., 1985, 1986; Whitaker and Patel, 1990; Wong et al., 2005). Ca^2+^ signals typically arise from a combination of Ca^2+^ release from intracellular stores, predominantly within the endoplasmic reticulum (ER), and Ca^2+^ entry across the plasma membrane. Store-operated Ca^2+^ entry, where loss of Ca^2+^ from the ER triggers opening of Ca^2+^ channels in the plasma membrane, is the most widely expressed Ca^2+^ entry pathway, but it is completely inhibited during mitosis (Smyth et al., 2009; Yu et al., 2019). Hence, mitotic Ca^2+^ signals are likely to be due entirely to release of Ca^2+^ from intracellular stores (Ciapa et al., 1994), most likely mediated by opening of inositol 1,4,5-trisphosphate receptors (IP_3_Rs), which are ubiquitously expressed intracellular Ca^2+^ channels (Rossi and Taylor, 2018). Vertebrates express three closely related IP_3_R subunits (IP_3_R1–3) that are differentially expressed and differ in both their sensitivity to IP_3_ and in their modulation by other signals, but each assembles to form homo- or hetero-tetrameric channels that open after IP_3_ binding (Alzayady et al., 2016). It is noteworthy that IP_3_Rs are phosphorylated by several mitosis-related kinases, including polo-like kinase 1 (PLK1), cyclin-dependent kinase 1 (CDK1), and extracellular signal-regulated kinases 1 and 2 (ERK1/2), each of which regulates responses to IP_3_ (Ito et al., 2008; Prole and Taylor, 2016; Sathanawongs et al., 2015). Furthermore, mitosis is accompanied by substantial subcellular redistribution of the ER and IP_3_Rs (Mitsuyama and Sawai, 2001; Parry et al., 2005).

We show that in cells without IP_3_Rs, the mitotic spindle fails to align properly. Although the NuMA crescent aligns appropriately in cells lacking IP_3_Rs, the spindle fails to align with that internal cue and astral microtubules around the daughter centrosome are shorter than those emanating from the mother centrosome. Expression of IP_3_R3 rescues spindle alignment, but only if the IP_3_R has a functional pore. We conclude that Ca^2+^ release by IP_3_Rs, which accumulate around centrosomes during metaphase and more so at the mother centrosome, is required for spindle alignment.

## Results

### IP_3_ Receptors Are Required for the Mitotic Spindle to Align with the Substratum

Human embryonic kidney (HEK) cells without IP_3_Rs (HEK-IP_3_R-KO cells) and wild-type (WT) cells grew at similar rates (Figure S1A), confirming that IP_3_Rs are not essential for proliferation (Ando et al., 2018; Atakpa et al., 2019; Sugawara et al., 1997). Time-lapse images of dividing cells showed that most WT cells formed a metaphase plate that aligned perpendicular to the substratum, allowing each daughter cell to remain attached to the substratum after cytokinesis (Figure S1B). This behavior is common to almost all epithelial cells and cell lines (Hehnly et al., 2015). However, many HEK-IP_3_R-KO cells formed a metaphase plate that was not perpendicular to the substratum, allowing only one daughter cell to remain attached, whereas the other was expelled into the medium (Figures S1C and S1D; Video S1). Imaging of cells expressing mCherry-α-tubulin and histone-2B-EGFP, to identify microtubules and chromosomes, respectively, showed that mitotic spindles in WT cells remained parallel to the substratum throughout metaphase, whereas, in HEK-IP_3_R-KO cells, the spindle rotated (Videos S2A and S2B). The misaligned spindles in HEK-IP_3_R-KO cells were often associated with aberrant mitoses, including nuclear division without cytokinesis (Video S2C), consistent with many cells having several nuclei or multipolar spindles (Figures S1E–S1H). The results are also consistent with a small interfering RNA (siRNA) screen, where loss of IP_3_R1 was associated with abnormal cytokinesis (Kittler et al., 2004). These observations prompted us to examine the relationship between IP_3_Rs and the mitotic spindle.

**Figure 1:**
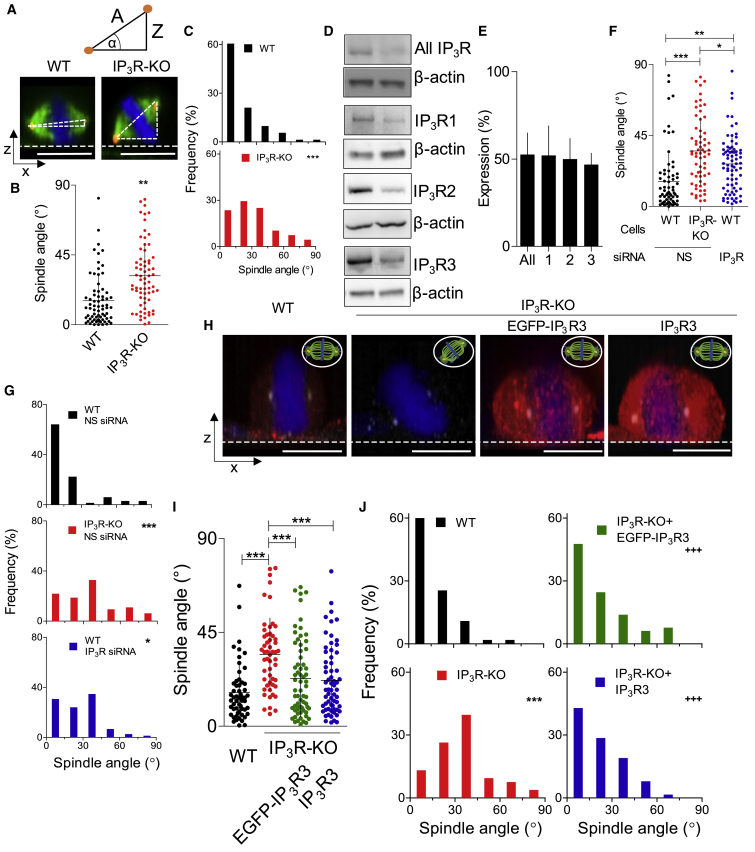
Loss of IP_3_Rs Causes Misalignment of Mitotic Spindles (A) Typical projections of confocal z stacks from cells stained for chromosomes (blue), α-tubulin (green), and γ-tubulin (red) show spindle angles (α) during metaphase for a WT and HEK-IP_3_R-KO cell. Dashed lines show substratum. Scale bars: 10 μm. (B) Spindle angles for WT and HEK-IP_3_R-KO cells (individual values, means ± SD from five experiments). ^∗∗^p < 0.01, Student’s t test. (C) Frequency distribution of spindle angles (n = 68–71 cells, from five experiments). ^∗∗∗^p < 0.001, χ^2^ test for trend. (D) Typical western blots (WBs) for IP_3_R subtypes in HEK cells treated with siRNAs to all three IP_3_R subtypes or non-silencing (NS) siRNA. (E) Summary results show IP_3_R expression determined by quantification of WB for cells treated with IP_3_R siRNA relative to NS siRNA (%, means ± SD, n = 4). (F) Spindle angles for matched comparisons of WT and HEK-IP_3_R-KO cells and cells treated with NS or IP_3_R siRNA (individual values, means ± SD from five experiments). ^∗^p < 0.05, ^∗∗^p < 0.01, ^∗∗∗^p < 0.001, ANOVA with Bonferroni test. (G) Frequency distributions of spindle angles for cells (WT or IP_3_R-KO) treated with NS or IP_3_R siRNA (n = 64–75 cells, from five experiments). ^∗∗∗^p < 0.001, ^∗^p < 0.05, relative to WT with NS siRNA, χ^2^ test for trend. (H) Cross-sections through 3D reconstructions of confocal z stack images of mitotic HEK cells expressing EGFP-IP_3_R3 or untagged IP_3_R3, immunostained for IP_3_R3 (red) and showing chromosomes (DAPI, blue) and γ-tubulin (white). Scale bars: 10 μm. (I) Spindle angles for WT and HEK-IP_3_R-KO cells transiently expressing IP_3_R3 or EGFP-IP_3_R3. Results (individual values, means ± SD, from five experiments). ^∗^p < 0.05, ^∗∗^p < 0.01, ANOVA with Bonferroni test. (J) Frequency distributions of spindle angles (n = 53–65 cells, from five experiments). ^∗∗∗^p < 0.001, relative to WT; ^+++^p < 0.001, relative to IP_3_R-KO, χ^2^ test for trend. See also Figures S1–S3 and Videos S1 and S2.

Video S1. Unstable Metaphase Plate and Out-of-Plane Division in HEK-IP_3_R-KO Cells, Related to Figure 1 Brightfield images of cells show a stable metaphase plate in the WT cell, but in the HEK-IP_3_R-KO cell the metaphase plate rotates and the cell then divides perpendicular to the substrate. Scale bars = 20 μm. Images captured at 3-min intervals and played at 7 frames/s (fps). Time shown as hr:min.

Video S2. Mitosis in HEK Cells with and without IP_3_R, Related to Figure 1 (A) Mitosis in WT HEK Cell. Widefield images of WT HEK cell expressing mCherry-α-tubulin (red) and histone 2B-EGFP (green). (B) Mitosis in HEK-IP_3_R-KO Cell. Similar analysis of HEK-IP_3_R-KO cell, showing rotation of the mitotic spindle in both horizontal and vertical planes. (C) Misaligned Mitotic Spindle in HEK-IP_3_R-KO Cell. Widefield images of HEK-IP_3_R-KO cell expressing mCherry-α-tubulin (red) and histone 2B-EGFP (green). Both poles of the spindle are evident at the beginning of the video (00:03), but as mitosis progresses one pole moves out of the focal plane (00:12 onwards), causing the spindle to misalign. In this example, nuclear division is not followed by cytokinesis, which causes the increased number of multinucleate cells in HEK-IP_3_R-KO cells (Figures S1E and S1F). Scale bars = 20 μm. Images captured at 1-min intervals and played at 7 fps. Times shown as hr:min.

We measured spindle angles relative to the substratum for semi-confluent HEK cells during metaphase (Figure 1A). In WT cells, most spindles aligned parallel to the substratum (a perpendicular metaphase plate), with an average spindle angle of 15.5° ± 16.4° (mean ± SD, n = 71 cells). Because the angles always have positive values, any variation causes the mean to deviate from the 0° that would indicate perfect alignment. In HEK-IP_3_R-KO cells, the bipolar spindles were more randomly oriented, evident from both the larger average spindle angle (31.6° ± 21.0°, n = 68) and the wider distribution of the angles (Figures 1B and 1C). Similar results were obtained using γ-tubulin to define spindle angles (Figure S1C) and from analyses of HAP1 cells with and without IP_3_Rs (Figures S2A–S2C).

Because the cell lines lacking IP_3_Rs were generated by selection after CRISPR/Cas9-mediated gene disruption (Alzayady et al., 2016; Atakpa et al., 2018), the misaligned spindles might have arisen through effects unrelated to the loss of IP_3_Rs. We, therefore, used siRNAs directed to each IP_3_R subtype to acutely reduce expression of all three IP_3_R subtypes in HEK cells. The siRNA treatment reduced expression of the IP_3_R subtypes similarly (Figures 1D and 1E). Spindle angles were significantly perturbed in siRNA-treated cells (26.9° ± 17.5°, n = 75), although less so than in HEK-IP_3_R-KO cells (35.2° ± 21.2°, n = 64) (Figure 1F). The distribution of spindle angles confirmed that loss of IP_3_Rs by siRNA or gene disruption had similar effects (Figure 1G).

HEK cells express all three IP_3_R subtypes (Mataragka and Taylor, 2018). In cells expressing only a single IP_3_R subtype, spindle angles were aberrant in cells expressing only IP_3_R1 (23.4° ± 14.7°, n = 72), but more normal in cells expressing only IP_3_R2 (18.1° ± 14.4°, n = 51) or IP_3_R3 (17.7° ± 13.9°, n = 66) (Figures S2D–S2F). We cannot conclude from these results that IP_3_R1 is incapable of contributing to spindle alignment because the HEK-IP_3_R1 cells may have acquired other defects during selection, but the results do establish that the requirement for IP_3_Rs can be satisfied by IP_3_R2 or IP_3_R3. Our subsequent studies focus on IP_3_R3 because plasmids encoding it are more reliably propagated than those expressing IP_3_R2, and there is a better antibody to IP_3_R3.

In HEK-IP_3_R-KO cells, the spindle angle (34.4° ± 17.4°, n = 53) was significantly rescued by expression of EGFP-IP_3_R3 (22.9° ± 18.3°, n = 65) to values that were not significantly different from the mock-transfected WT cells (16.2° ± 13.4°, n = 55) (Figures 1H–1J). We confirmed that the EGFP tag did not affect IP_3_R3 function by demonstrating that, for matched levels of IP_3_R3 expression (and comparable to WT cells), rescue of spindle alignment was indistinguishable for EGFP-IP_3_R3 (22.9° ± 18.3°) and untagged IP_3_R3 (22.0° ± 16.4°, n = 63 cells) (Figures 1H–1J and S3). Furthermore, expression of IP_3_R3 at levels substantially exceeding native levels did not perturb spindle alignment (Figure S3A). In WT cells, however, endogenous IP_3_R3 expression inversely correlated with spindle angle, suggesting that native levels of IP_3_R expression may be limiting for appropriate spindle alignment (Figure S3A). The results so far establish that IP_3_Rs are required for the mitotic spindle to align with the substratum and that IP_3_R3 (or EGFP-IP_3_R3) is sufficient to meet that need.

### IP_3_ Receptors Are Required to Align Spindles with the NuMA Complex

Spindle angles were measured relative to the substratum (Figures 1A and S1B) using semi-confluent cells, in which most cells contact at least one neighbor. We therefore considered whether spindle misalignment in cells without IP_3_Rs arose from ineffective decoding of appropriate intracellular signals by the spindle apparatus or from ineffective detection of external cues, for example β1-integrin-mediated interaction with the extracellular matrix (Iwano et al., 2015) or cadherin-mediated interactions between cells (Hart et al., 2017).

**Figure 2:**
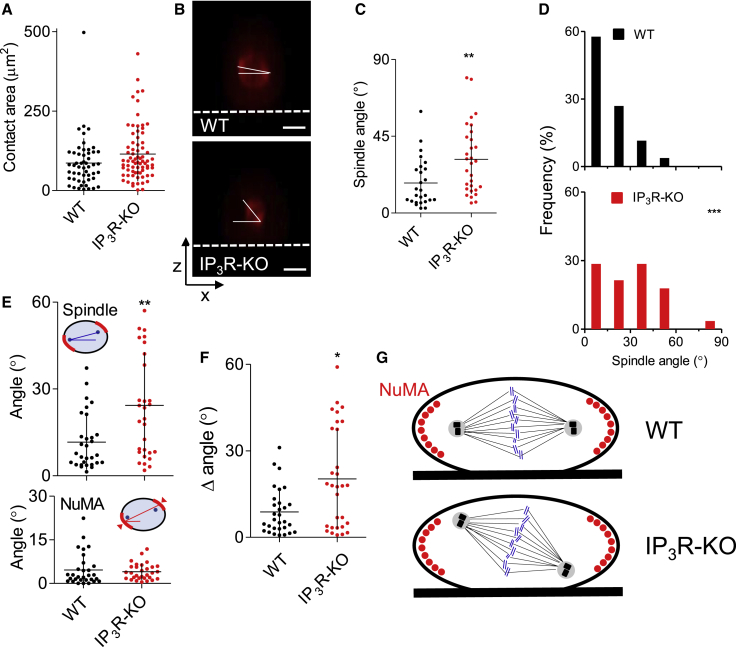
IP_3_Rs Are Required for Spindles to Align with Intracellular Cues (A) Contact areas between PM and substratum (individual values from five experiments, means ± SD). p > 0.05, Student’s t test. From Figure S4A. (B) Side view of confocal z stacks showing spindle angles identified by mCherry-α-tubulin in isolated cells. Dashed lines show substratum. Scale bars: 10 μm. (C) Spindle angles in isolated synchronized HEK cells with no intercellular contacts, from (B). Individual values, means ± SD from three experiments. ^∗∗^p < 0.01, Student’s t test. (D) Frequency distribution of spindle angles in isolated cells (n = 26–28 cells, from three experiments). ^∗∗∗^p < 0.001, χ^2^ test for trend. (E) Spindle (from γ-tubulin staining, top) and NuMA angles (bottom) in WT and HEK-IP_3_R-KO cells, each measured relative to the substratum. Individual values, means ± SD from four experiments. ^∗∗^p < 0.01, Student’s t test. (F) Difference (Δ) between spindle and NuMA angles for individual cells. Individual values, means ± SD, n = 4 experiments. ^∗^p < 0.05, Student’s t test. (G) NuMA aligns normally in IP_3_R-KO cells, but centrosomes no longer align appropriately with NuMA. See also Figures S4A–S4E.

Using synchronized dividing cells stained with a lipid marker, we confirmed that HEK-IP_3_R-KO cells adhere normally and expose similar areas of plasma membrane (PM) to the substratum as WT cells (Figures 2A and S4A). In sparsely distributed synchronized WT cells expressing mCherry-α-tubulin, spindles aligned with the substratum (17.6° ± 14.1°, n = 26) (Figures 2B and S4B), consistent with published results from isolated HeLa cells (Toyoshima et al., 2007), but again, the spindles were misaligned in HEK-IP_3_R-KO cells (31.4° ± 20.3°, n = 30) (Figures 2B–2D). We conclude that misaligned spindles in cells without IP_3_Rs are not due to defects in either attachment to the substratum or communication between cells.

In vertebrates, NuMA is released from the nucleus during nuclear envelope breakdown. NuMA, associated with the microtubule motor dynein, then forms a complex at the PM to orient the spindle poles (Bergstralh et al., 2017; Du and Macara, 2004; Gloerich et al., 2017) (Figure S4C). NuMA expression (99.0% ± 9.9%, n = 3, determined by western blot [WB]) and the lengths of NuMA crescents (19.3 ± 8.0 μm and 21.0 ± 7.2 μm) were indistinguishable in WT and IP_3_R-KO cells. We investigated whether the need for IP_3_Rs arose upstream or downstream of NuMA complexes by measuring spindle angles and angles between the centers of NuMA crescents in the same metaphase cells. Spindles were misaligned in HEK-IP_3_R-KO cells (24.3° ± 18.4° versus 11.6° ± 9.6° in WT cells, n > 30), but the NuMA angles were indistinguishable in cells with (4.6° ± 5.5°, n = 30) and without (4.0° ± 2.8°, n = 30) IP_3_Rs (Figures 2E, S4D, and S4E). The disjunction of NuMA and the spindle in cells without IP_3_Rs is clear from comparison of the difference in their angles within individual cells (Δ = 8.23° ± 7.96° and 20.2° ± 17.5° in WT and IP_3_R-KO cells, respectively, n = 30) (Figure 2F). These results suggest that IP_3_Rs are not required to align NuMA with the substratum, but they are required for the spindle to align with the NuMA complex (Figure 2G).

**Figure 3:**
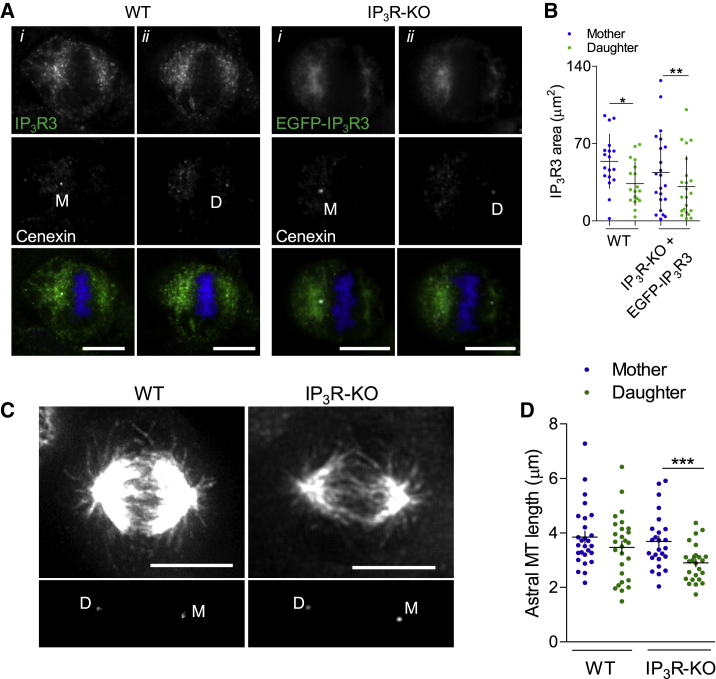
IP_3_R-Evoked Ca^2+^ Signals Are Required for Spindle Alignment (A) Confocal images of metaphase WT cells or HEK-IP_3_R-KO cells expressing EGFP-IP_3_R3, immunostained for IP_3_R3 (for WT) and cenexin (white, to distinguish mother [M] and daughter [D] centrosomes). Images show sections in which the mother (*i*) or daughter (*ii*) centrosome was most intensely stained. Overlay images show IP_3_R (green, EGFP or immunostaining), cenexin (white), and DNA (DAPI, blue). Scale bars: 10 μm. (B) Summary results (individual values, means ± SD, from three experiments) show areas populated by IP_3_R3 surrounding the two centrosomes (immunostaining or EGFF). ^∗^p < 0.05, ^∗∗^p < 0.01, paired Student’s t test for mother relative to daughter. (C) Z stacks of five confocal sections show astral microtubules (α-tubulin staining) and mother (M) and daughter (D) centrosomes (cenexin staining). Scale bars: 10 μm. (D) Summary results (individual values, means ± SEM) show mean lengths of astral microtubules in individual cells associated with mother and daughter centrosomes (typically four measurements for each centrosome in each cell). ^∗∗∗^p < 0.001, Student’s t test. See also Figure S5.

Loss of IP_3_Rs had no significant effect on the mean length of the astral microtubules measured in z stacks of confocal images (Figure S5A). The mother centrosome, which contains the oldest centrosome, more effectively nucleates microtubules, forms more extensive astral microtubules during mitosis, contributes to cell polarity, and can affect cell fate (Hehnly et al., 2015; Yamashita, 2009). In WT HEK cells and in HEK-IP_3_R-KO cells expressing EGFP-IP_3_R3 (wherein the distribution of IP_3_R3 mimics that of WT cells; Figure S5B), IP_3_R3 concentrated most around the mother centrosome during metaphase (Figures 3A and 3B). Indistinguishable results were obtained using immunostaining (WT cells) and EGFP-fluorescence (HEK-IP_3_R-KO cells expressing EGFP-IP_3_R3) (Figure 3B). These observations prompted us to determine separately the length of astral microtubules around mother and daughter centrosomes. The results indicate that in HEK-IP_3_R-KO cells, the astral microtubules around the daughter centrosome are shorter than those around the mother centrosome (Figures 3C, 3D, S5C, S5D, S5F, and S5G). There were no significant differences between cells with and without IP_3_Rs in the length of the mitotic spindle (9.44 ± 1.27 μm, n = 53 in WT cells, and 9.99 ± 1.41 μm, n = 57 in HEK-IP_3_R-KO cells, mean ± SD), the distance between the cortex and mother centrosome (5.36 ± 2.35 μm, n = 26 and 4.86 ± 2.85 μm, n = 25) or daughter centrosome (5.81 ± 2.57 μm and 4.55 ± 2.02 μm), the fraction of the plus ends of microtubules (identified with end-binding protein 3, EB3) abutting the cortex around the mother or daughter centrosome (Figures S5C–S5E), or the density of EB3 puncta around the centrosomes (Figure S5H)

Collectively, these results establish that IP_3_Rs are required for centrosomes to align appropriately with internal cues provided by the NuMA complex and that astral microtubules radiating from mother and daughter centrosomes are differentially affected by the loss of IP_3_Rs.

### Ca^2+^ Release by IP_3_ Receptors Is Required for Spindle Alignment

**Figure 4:**
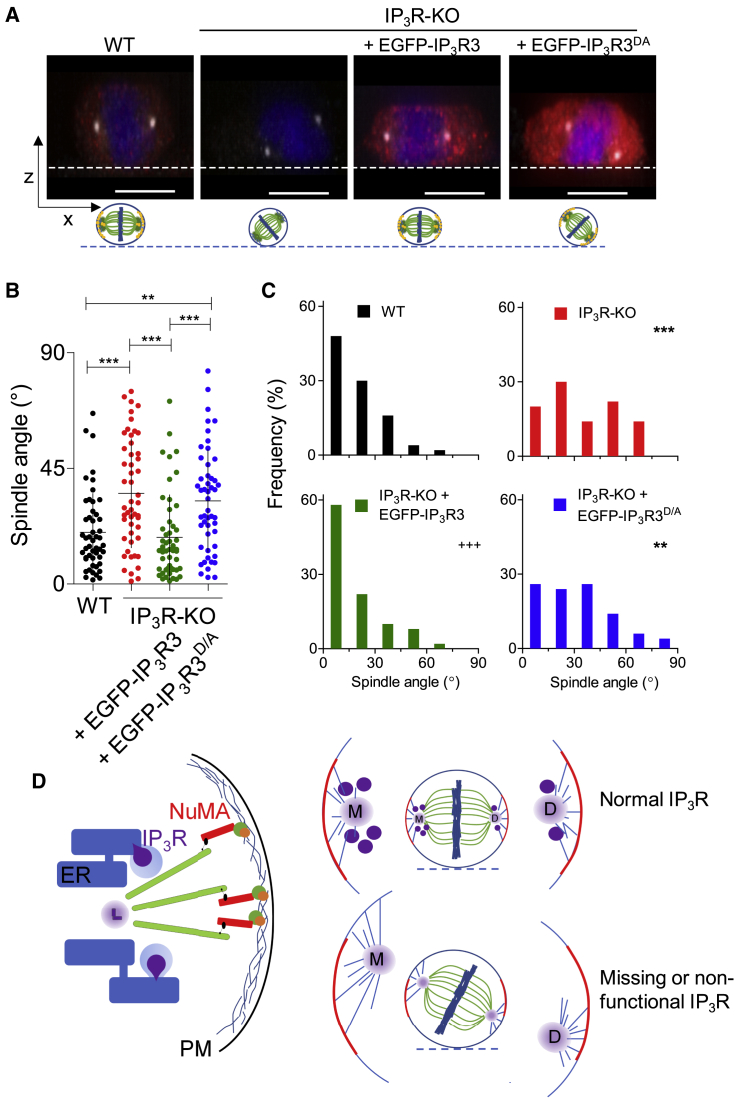
Ca^2+^ Signals Evoked by IP_3_Rs Are Required for Spindle Alignment (A) Side views of 3D-projected z stacks from metaphase cells immunostained for IP_3_R3 (red) and γ-tubulin (gray) and with DNA stained with DAPI (blue). Dashed lines show substratum. Scale bar: 10 μm. (B) Spindle angles (individual values, means ± SD, n = 50 cells from five experiments). ^∗∗∗^p < 0.001, ^∗∗^p < 0.01, ANOVA with Bonferroni test. (C) Frequency distribution of spindle angles (n = 50 cells from five experiments for each condition). ^∗∗∗^p < 0.001, ^∗∗^p < 0.01, relative to WT; ^+++^p < 0.001, relative to IP_3_R-KO, χ^2^ test for trend. (D) During metaphase, IP_3_Rs (purple circles) accumulate around the centrosomes, more so around the mother (M) than the daughter (D) centrosome. Astral microtubules, through their association with NuMA and cortical actin, control orientation of the mitotic spindle. IP_3_R-evoked Ca^2+^ signals, by regulating astral microtubules, influence spindle orientation. In cells without functional IP_3_Rs, astral microtubules around the mother centrosome are longer and the spindles less effectively align with NuMA. See also Figures S4F–S4H.

IP_3_Rs are best known for releasing Ca^2+^ from the ER (Rossi and Taylor, 2018), but additional proteins associate with IP_3_Rs (Prole and Taylor, 2016), and many IP_3_Rs appear not to release Ca^2+^ in intact cells (Thillaiappan et al., 2017). We, therefore, considered whether Ca^2+^ release through IP_3_Rs was required for spindle alignment. Mutation of a single residue within the pore of IP_3_R1 prevents it from conducting Ca^2+^ (Boehning et al., 2001; Dellis et al., 2008). We mutated the equivalent residue (D2477A) in EGFP-IP_3_R3 (EGFP-IP_3_R3^D/A^) and confirmed that it prevented IP_3_ from evoking Ca^2+^ release (Figures S4F–S4H). Expression of EGFP-IP_3_R3 in HEK IP_3_R-KO cells rescued spindle alignment, and the similar lengths of astral microtubules at mother and daughter centrosomes were restored, but comparable expression of EGFP-IP_3_R3^D/A^ rescued neither feature (Figures 4A–4C, S5F, and S5G). These results establish that Ca^2+^ release through IP_3_Rs is required for mitotic spindles to align properly (Figure 4D).

## Discussion

Progression through the cell cycle is controlled by regulated degradation of cyclins, but local Ca^2+^ signals are also important at critical stages (Keith et al., 1985; Parry et al., 2005; Zhao et al., 2019), including nuclear envelope breakdown, the transition from metaphase to anaphase and cytokinesis. The mechanisms are largely unresolved, but because store-operated Ca^2+^ entry is inhibited during meiosis and mitosis (Smyth and Putney, 2012; Yu et al., 2019), the Ca^2+^ signals are probably evoked by IP_3_Rs. We have shown that cells can proliferate without IP_3_Rs (Figure S1A) (Alzayady et al., 2016; Ando et al., 2018; Atakpa et al., 2019; Sugawara et al., 1997), but their behavior is compromised. In some cells without IP_3_Rs, the mitotic spindle is aberrant and cytokinesis sometimes fails (Figure S1). However, most cells divide successfully but have spindles that fail to align with the substratum because they no longer associate appropriately with the NuMA complex (Figures 1 and 2). The defects are reversed by expression of IP_3_R3, but only if it has a functional Ca^2+^ pore (Figure 4). This is consistent with recent work suggesting that local Ca^2+^ signals occur near centrosomes throughout mitosis, and abolishing them at one centrosome perturbs mitosis (Helassa et al., 2019). It is noteworthy that variations in expression of endogenous IP_3_Rs are associated with the reliability of spindle alignment: WT cells with fewer IP_3_Rs are more likely to have misaligned spindles (Figure S3A).

Astral microtubules link the NuMA complex to the pericentriolar material (PCM) that surrounds each centrosome (Figure S4C). IP_3_Rs concentrate most around the mother centrosome (Figures 3A and 3B), which nucleates astral microtubules that are more abundant and longer than those associated with the daughter centrosome (Hehnly et al., 2015; Yamashita, 2009). There is a significant disparity in the length of astral microtubules emanating from the mother and daughter centrosomes in cells without IP_3_Rs: astral microtubules at the mother pole are longer than those at the daughter pole (Figures 3C, 3D, S5F, and S5G), although they appear to make similar numbers of contacts with the cortex at both poles in cells with or without IP_3_Rs (Figures S5C–S5E). Our results indicate an asymmetric relationship between IP_3_Rs and the mother and daughter centrosomes. The mother centrosome has greater capacity to nucleate microtubules and associates with additional proteins (Huhn et al., 2017) and IP_3_Rs concentrate most around it (Figures 3A and 3B), and in cells without IP_3_Rs, there is a relative lengthening of astral microtubules around the mother centrosome (Figures 3C, 3D, S5F, and S5G). We speculate that the asymmetric lengths of astral microtubules around mother and daughter centrosomes in cells without IP_3_Rs may generate uneven forces between them and the cortex (Howard and Garzon-Coral, 2017), leading to spindle misalignment.

We conclude that Ca^2+^ release through IP_3_Rs is required for the mitotic spindle to align appropriately, probably through regulation of astral microtubules by IP_3_R-evoked Ca^2+^ signals (Figure 4D). Future experiments should assess whether the Ca^2+^ signals that occur during metaphase (Helassa et al., 2019) regulate microtubules and their motors or deliver Ca^2+^ locally to the mitochondria to allow local delivery of ATP (Zhao et al., 2019) and thereby regulation of microtubule activity. Our results reveal that IP_3_R-evoked Ca^2+^ signals are an important means of regulating the mitotic spindle.

## STAR★Methods

### Key Resources Table

**Table:** 

REAGENT or RESOURCE	SOURCE	IDENTIFIER
**Antibodies**		

**WB, western blot; IC, immunocytochemistry**		

Mouse monoclonal anti-α-tubulin (IC, 1:1000)	Sigma Aldrich, Gillingham, UK	cat# T6074; RRID: AB_477582
Rabbit anti-γ-tubulin (IC, 1:400)	GeneTex, CA, USA	cat# GTX113286; RRID: AB_1952442
Rabbit anti-cenexin (aka ODF2) (IC, 1:200-400)	GeneTex	cat# GTX114594; RRID: AB_2814951
Mouse monoclonal anti-β-actin (WB, 1:5000)	Cell Signaling Technology, Danvers, MA, USA	cat# 3700; RRID: AB_2242334
Mouse monoclonal anti-IP_3_R1 (WB, 1:1000)	Abclonal, Tokyo, Japan.	cat# A7905; RRID: AB_2770019
Rabbit anti-IP_3_R2 (WB, 1:1000)	Pocono Rabbit Farm & Laboratory, Inc., Canadensis, PA, USA	Custom-made for our laboratory (Mataragka and Taylor, 2018)
Mouse monoclonal anti-IP_3_R3 (WB, 1:1000; IC, 1:200)	BD Bioscience, NJ, USA	cat# 610313; RRID: AB_397705
Rabbit anti-IP_3_R (recognizes all IP_3_R subtypes) (WB, 1:1000; Figure 1D).	Pocono Rabbit Farm & Laboratory, Inc.	Custom-made for our laboratory (Mataragka and Taylor, 2018)
Rabbit anti-IP_3_R (recognizes all subtypes) (WB, 1:1000; Figure S2D). Although described as anti-IP_3_R1, the antigenic peptide is common to all three IP_3_R subtypes.	Cell Signaling Technology	cat# 8568; RRID: AB_10890699
Rat monoclonal anti-GFP (WB, 1:2000)	Chromotek, Munich, Germany	cat# 3h9-100; RRID: AB_10773374
Rat monoclonal anti-EB3 antibody (IC, 1:100)	AbCam, Cambridge UK.	cat# Ab53360; RRID AB_880026
IgG-HRP (WB, 1:5000)	Santa Cruz Biotechnology	cat# sc-516102; RRID: AB_2687626
Mouse anti-rabbit IgG-HRP (WB 1:5000)	Santa Cruz Biotechnology	cat# sc-2357; RRID: AB_628497
Goat anti-rat IgG-HRP (WB, 1:5000)	Santa Cruz Biotechnology	cat# sc-2006; RRID: AB_1125219
Alexa Fluor 647 goat anti-rabbit IgG (IC, 1:400)	ThermoFisher	cat# A21244; RRID: AB_141663
Alexa Fluor 488 goat anti-mouse IgG (IC, 1:400)	ThermoFisher	cat# A10667; RRID: AB_2534057
Alexa Fluor 568 goat anti-mouse IgG (IC, 1:400)	ThermoFisher	cat# A11031; RRID: AB_144696
Alexa Fluor 488 goat anti-rat IgG (IC, 1:100)	Thermofisher	cat# A11006; RRID: AB_141373
Alexa Fluor 568 goat anti-rat IgG (IC, 1:100)	Thermofisher	cat# A11077; RRID: AB_141874
Goat anti-rabbit IgG conjugated to CF405M (IC, 1:100)	Sigma	cat# SAB4600461
GFP-Booster ATTO488 nanobody (IC, 1:500)	Chromotek	cat# gba488-100; RRID: AB_2631386

**Chemicals, Peptides, and Recombinant Proteins**		

Bovine serum albumin (BSA)	Europa Bio-Products, Ely, UK	cat# EQBAH64
Calbryte 590 AM	AAT Bioquest, Sunnyvale, CA, USA	cat# 20700
Carbamoylcholine chloride (carbachol, CCh)	Sigma Aldrich	cat# C-4382
cOmplete, EDTA-free protease inhibitor cocktail	Roche	cat# 11836153001
4′,6-Diamidino-2-phenylindole dihydrochloride (DAPI)	Sigma Aldrich	cat# D9542
3,3′-Dihexyloxacarbocyanine iodide (DHCC)	Sigma Aldrich	cat# 318426
Dulbecco’s Modified Eagle’s Medium (DMEM)/F-12 with GlutaMAX	ThermoFisher	cat# 31331093
ECL Prime western blotting detection reagent	Amersham	cat# RPN2236
Fibronectin (human)	Merck Millipore, Watford, UK	cat# FC010
Fetal bovine serum (FBS)	Sigma Aldrich	cat# 094N3341
GIBCO TrypLE Express	ThermoFisher	cat# 12605010
HEPES	Merck Millipore	cat# 391338
HiPerFect	QIAGEN, Hilden, Germany	cat# 301705
Ionomycin	Cambridge Bioscience	cat# CAYM11932
Iscove’s Modified Dulbecco’s Medium (IMDM)	ThermoFisher	cat# 12440-053
NucBlue Live ReadyProbes reagent	ThermoFisher	cat# R37606
PVDF iBlot transfer stack	ThermoFisher	cat# IB401031
Run Blue (4-12%) SDS gel	Expedeon, San Diego, CA, USA	cat# NXG40812
Thymidine	Sigma Aldrich	cat# T9250
TransIT-LT1 reagent	MirusBio, WI, USA	cat# MIR 2300
Tris	ThermoFisher	cat# BP152-1
Tween-20	Sigma-Aldrich	cat# T5927
Trypan Blue	ThermoFisher	cat# T10282
Vectashield	VectorLabs, Burlingame, CA, USA	cat# H-1000
Wheat germ agglutinin (WGA)-CF405M	Biotium, CA, USA	cat# 29028-1
Wheat germ agglutinin (WGA)-CF568	Biotium	cat# 29077-1

**Critical Commercial Assays**		

QuickChange Lightning site-directed mutagenesis kit	Agilent, Santa Clara, CA USA.	cat# 210515

**Experimental Models: Cell Lines**		

HEK cells	Dr D. Yule (University of Rochester, NY, USA)	Dr D. Yule (University of Rochester, NY, USA)
HEK-IP_3_R-KO cells	Kerafast, Boston, MA, USA	cat# EUR030
HEK-IP_3_R1 cells	Kerafast	cat# EUR031
HEK-IP_3_R2 cells	Kerafast	cat# EUR032
HEK-IP_3_R3 cells	Kerafast	cat# EUR033
HAP1 cells	Horizon Discovery, Cambridge, UK	cat# C631
HAP1-IP_3_R-KO cells	(Atakpa et al., 2018)	Available from Horizon Discovery

**Oligonucleotides**		

IP_3_R1 siRNA	QIAGEN	cat# Hs_ITPR1_4 FlexiTube siRNA (SI00034545)
IP_3_R2 siRNA	QIAGEN	cat# Hs_ITPR2_1 FlexiTube siRNA (SI00034552)
IP_3_R3 siRNA	QIAGEN	cat# Hs_ITPR3_1 FlexiTube siRNA (SI00034580)
Non-silencing (NS) siRNA	QIAGEN	cat# 1027281

**Recombinant DNA**		

pcDNA3.2/DEST-EGFP-IP_3_R3 (rat)	(Pantazaka and Taylor, 2011)	N/A
pcDNA3.2/V5DEST-IP_3_R3 (rat)	(Tovey et al., 2010)	N/A
pShuttle mCherry-α-tubulin	Addgene	Addgene plasmid # 26768; RRID: Addgene_26768 (Matov et al., 2010)
Histone 2B-EGFP	Addgene	Addgene plasmid # 11680; RRID: Addgene_11680 (Kanda et al., 1998)
GFP-ER		(Woźniak et al., 2009)

**Software and Algorithms**		

GraphPad Prism 6.0	GraphPad Software, 6.0	https://www.graphpad.com/
Excel	Microsoft, 2007	N/A
FIJI		https://fiji.sc/
GeneTools, version 4	Syngene, Cambridge, UK	https://www.syngene.com/
MetaMorph Microscopy Automation and Image Analysis	Molecular Devices, San Jose, CA	https://www.moleculardevices.com

### Resource Availability

#### Lead Contact

Further information and requests for resources and reagents should be directed to, and will be fulfilled by, the Lead Contact, Colin W Taylor (cwt1000@cam.ac.uk).

#### Materials Availability

This study did not generate any unique materials. All materials needed to support the claims of the study are available commercially.

#### Data and Code Availability

This study did not generate any unique dataset or code that is required to support the claims of the paper.

### Experimental Model and Subject Details

We used two cell lines, human embryonic kidney (HEK) 293 cells and HAP1 cells (near-haploid) and their derivatives. At the time of the study, these were the only mammalian cell lines in which all three IP_3_R subtypes had been disrupted. HEK293 cells are hypotriploid cells derived from embryonic kidney, immortalized by transformation with adenovirus, and with features suggesting derivation from a neural lineage (Shaw et al., 2002). The four HEK cell lines used (HEK-IP_3_R-KO or expressing only one of the three IP_3_R subtypes) were generated in Dr D. Yule’s laboratory using CRISPR/Cas9 (Alzayady et al., 2016). The edited cell lines were supplied by Kerafast (Boston, MA, USA), and the parental cell line (WT), from which the edited cells were generated, was provided by Dr Yule (University of Rochester, NY, USA). HAP1 cells are human, near-haploid, fibroblast-like cells derived from the chronic myelogenous leukemia (CML) cell line, KBM-7. The cell line in which genes encoding all three IP_3_R subtypes were disrupted by CRISPR/Cas9 (HAP1 IP_3_R-KO) has been described previously (Atakpa et al., 2018). We have not independently verified the authenticity of the cell lines.

### Method Details

#### Cell Culture and Transfection

HEK cells were cultured in DMEM/F-12 with GlutaMAX and fetal bovine serum (FBS, 10%) at 37°C in humidified air with 5% CO_2_. HAP1 cells were cultured in IMDM with FBS (10%) at 37°C in humidified air with 5% CO_2_. Cells were passaged every 3-4 days using GIBCO TrypLE Express. Regular screening confirmed that all cells were free of mycoplasma.

For imaging, cells were grown on 16-mm round glass coverslips (N^o^ 0, VWR, International, Radnor, PA, USA) or 35-mm glass-bottomed imaging dishes (#P35G-1.0-14-C, MatTek Corporation, Ashland, MA, USA) coated with human fibronectin (50 μg/mL).

Transient transfection with pcDNA3.2/V5DEST-IP_3_R3, pcDNA3.2/DEST-EGFP-IP_3_R3, pcDNA3.2/DEST-EGFP-IP_3_R3^D/A^, pShuttle mCherry-α-tubulin or H2B-EGFP plasmids used TransIT-LT1 reagent (1 μg DNA/3 μL reagent) according to the manufacturer’s instructions. Transfection with siRNA against each IP_3_R subtype (40 nM of each) or a non-silencing (NS) siRNA (120 nM) used HiPerfect transfection reagent according to the manufacturer’s instructions. Cells were used after 48 hr. pcDNA3.2/DEST-EGFP-IP_3_R3 was used to modify the coding sequence of EGFP-IP_3_R3 from D2477 (CGA) to A (GCC) using primers 5′-TGCGGAGGATGGCGCCCACGCCG-3′ and 5′-CGGCGTGGGCGCCATCCTCCGCA-3′ and the QuickChange Lightning site-directed mutagenesis kit according to the manufacturer’s instruction. Sequencing of the entire coding sequence confirmed the single mutation.

Most experiments used non-synchronized cells, but to obtain sufficient cells in a field for analyses of isolated cells, cells were synchronized using either a single- or double-thymidine block. Thymidine arrests cells at the G1/S boundary by inhibiting DNA synthesis, and cells then synchronously enter S phase when thymidine is removed. For the single-block, HEK cells (10^5^/well) grown on 35-mm imaging dishes coated with human fibronectin (50 μg/mL) were incubated with thymidine (2 mM) for 18-24 hr, washed three times in phosphate-buffered saline (PBS) and incubated in fresh medium for 7-10 hr before analysis. For the double-block, cells were incubated with thymidine (2 mM, 18 hr), washed, incubated in thymidine-free medium (9 hr), incubated again with thymidine (2 mM, 18 hr), washed and incubated in fresh medium (8-10 hr). We used these methods to avoid synchronization protocols that rely on perturbation of microtubules.

A Countess automated cell counter (ThermoFisher) was used to count cells in medium containing 0.2% Trypan Blue.

#### Immunocytochemistry

Cells (10^5^/well) grown (24 hr) on 16-mm coverslips were fixed in ice-cold methanol, permeabilized in Tris-buffered saline (TBS: 50 mM Tris-HCl, 150 mM NaCl, pH 7.5) with Tween-20 (0.1%) and Triton X-100 (0.1%), and then blocked in TBS containing Tween (0.1%) and BSA (2%). Cells were incubated with primary antibody (1 hr, 20°C), washed three times with TBS containing 0.1% Tween, and incubated with fluorescent secondary antibodies (1 hr, 20°C). DAPI (300 nM, 10 min, 20°C) was used to stain nuclei. Fluorescently tagged WGA (5 μM, 10 min 37°C, added before fixing cells) was used to identify the plasma membrane. Coverslips were mounted with Vectashield on microscope slides over two stripes of nail polish, sealed with nail polish, and observed using an Olympus IX83 microscope with 60x and 100x objectives (numerical apertures, NA, 1.3 and 1.49 respectively). For total internal reflection fluorescence microscopy (TIRFM) the penetration depth was 90-140 nm. An iLas2 illumination system (Cairn, Faversham, Kent, UK) was used for TIRFM and wide-field imaging. For quantification of IP_3_R3 immunostaining (Figures S3A and S3B), we used the background-corrected complete z stack from confocal images.

#### Live-Cell Imaging

Cells transfected to express mCherry-α-tubulin alone or with H2B-EGFP, and grown in imaging dishes were washed and incubated in HEPES-buffered saline (HBS: 135 mM NaCl, 5.9 mM KCl, 1.2 mM MgCl_2_, 1.5 mM CaCl_2_, 11.5 mM glucose, 11.6 mM HEPES, pH 7.4) containing FBS (10%). Wide-field images of cells in HBS were collected at 1-min intervals using an Olympus IX83 microscope with a 100x objective (NA, 1.49) within an enclosed cabinet (37°C, 5% CO_2_) using 488/525 nm (excitation laser/emission filter) and 561/630 nm lasers, and an iLas2 targeted laser illumination system.

#### Measurement of Contact Areas Between Cells and Substratum

Synchronized HEK cells were incubated in HBS with 3,3′-dihexyloxacarbocyanionie iodide (DHCC, 20 μg/mL, ~1 min). Live cells were imaged within 20 min by TIRFM and confocal microscopy (488/525 nm) using an Olympus IX83 microscope with 100x objective (NA, 1.49) to determine the areas contacting the substratum and across a mid-section of each cell.

#### Measurements of Cytosolic Ca^2+^ Signals

HEK cells in HBS were incubated with Calbryte 590-AM (5 μM, 1 hr, 20°C in darkness), washed and incubated in HBS (45 min, 20°C) before imaging. Wide-field fluorescence images (488/525 nm to detect EGFP, 561/630 nm to detect Calbryte 590) were collected at 500-ms intervals using an Olympus IX83 microscope with a 100x objective (NA, 1.49). After subtraction of background fluorescence (from an area outside the cell), changes in fluorescence (F/F_0_) are reported relative to basal fluorescence (F_0_).

#### Western Blots

Cells were scraped into cold RIPA medium (1 mM Tris HCl, 15 mM NaCl, 0.5 mM EDTA, 0.1% Triton X-100, pH 7.5) containing protease inhibitors (cOmplete, EDTA-free protease inhibitor cocktail) and lysed using a syringe and 18G needle. The protein content of the supernatant (10,000 x*g*, 10 min) was quantified using a Bradford assay with BSA as standard. Proteins were separated using 4%–12% Run Blue Bis-tris gels, transferred to a polyvinyl difluoride (PVDF) membrane using an iBlot gel-transfer system, blocked (1 hr, 20°C) in TBS with Tween-20 (0.1%) and BSA (5%), and incubated (1 hr, 20°C) with primary antibody in fresh blocking buffer. After three washes, the membrane was incubated with HRP-conjugated secondary antibody (1 hr, 20°C) and washed three times. Bands were visualized with ECL Prime western blotting detection reagent using a GeneTools Syngene PXi chemiluminescence detection system, and quantified using FIJI (after subtracting a background measured from an area adjacent to the relevant band). Band intensities were expressed relative to control bands on the same gel.

### Quantification and Statistical Analysis

#### Measurements of Angles of Mitotic Spindles and NuMA Crescents

We used a published method to measure spindle angle relative to the substratum (Toyoshima et al., 2007; Toyoshima and Nishida, 2007). Briefly, confocal z stack images (0.5-μm thick) of samples stained for α- or γ-tubulin, or transfected with mCherry-α-tubulin, were projected as a 3D image to obtain a side view using FIJI software 3D project plugin. The distance between the mitotic poles (A) and the height difference between them (Z) was measured (spindle angle = ArcSin(Z/A)) (Figure 1A).

For NuMA crescent angles, the difference between the centers of each crescent in a z stack (Z_1_-Z_2_) and their separation in the x-plane (X) were measured (NuMA angle = ArcTan((Z1-Z2)/X)) (Figure S4E).

#### Measurements of IP_3_R Distributions Around Mother and Daughter Centrosomes

Mother and daughter centrosomes were distinguished by immunostaining for cenexin, which more intensely labels the mother centrosome (Colicino et al., 2019). The confocal section in which each centrosome was most intensely labeled was then used to analyze IP_3_R distribution (immunostained or EGFP) around that centrosome. For each section, the average fluorescence intensity (mean ± SD) was determined, and pixels surrounding each centrosome that exceeded a threshold (mean + 2SD) were used to calculate areas occupied by IP_3_Rs (Figure 3B).

#### Measurements of Lengths of Astral Microtubules

We used two different methods to measure the length of astral microtubules: tracing anti-α-tubulin staining from each centrosome (Figures 3C and 3D) and measuring distances from centrosomes to immunostained EB3 (Figures S4C–S4G). Cells immmunostained for cenexin, α-tubulin and/or EB3 and/or with the plasma membrane identified by WGA-staining were imaged using spinning-disk confocal microscopy. After background correction, Z stack projections of five sections that included each centrosome were used to measure the lengths of astral microtubules in the area generated by projecting a line passing through each centrosome, perpendicular to the spindle axis using FIJI (Figure S4E). The same methods were used to quantify cortical EB3 by counting immunostained EB3 puncta associated with WGA.

#### Statistical Analyses

Statistical analyses used GraphPad Prism version 6. Results are presented as means ± SD or SEM, as appropriate. Paired or unpaired Student’s t tests (for 2 variables) or one-way ANOVA with Bonferroni post hoc test (for multiple comparisons) was used for statistical analyses (^∗^p < 0.05, ^∗∗^p < 0.01 and ^∗∗∗^p < 0.001). χ^2^ test for trend was used for comparisons of frequency distributions. Sample sizes and the tests used are provided in figure legends.

## Supplemental Information

Document S1. Figures S1–S5

Document S2. Article plus Supplemental Information
