# Supplementary material for: Ca2+ Release by IP3 Receptors Is Required to Orient the Mitotic Spindle
Source: Cell Rep. 2020 Dec 15;33(11):108483. doi: 10.1016/j.celrep.2020.108483 (PMC7758162; doi:10.1016/j.celrep.2020.108483)
Supplement: Document S1. Figures S1–S5 [file mmc1.pdf]

Cell Reports, Volume *33*

Supplemental Information

Ca^{2+} Release by IP_3 Receptors Is Required to Orient the Mitotic Spindle

Raul Lagos-Cabré, Adelina Ivanova, and Colin W. Taylor

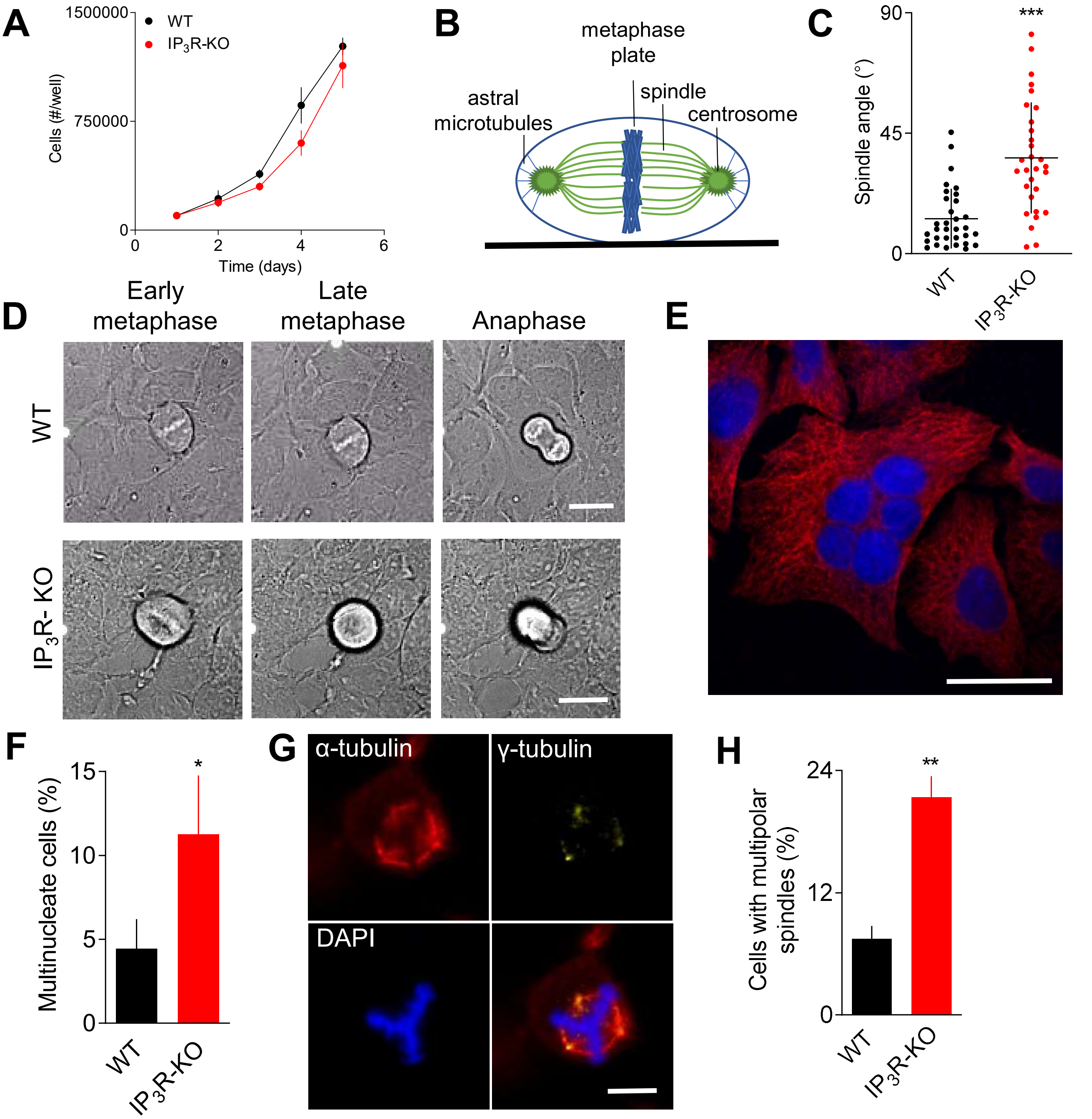

Figure S1. Cells Without IP$_3$Rs Proliferate Normally But With Aberrant Spindles. Related to Figure 1.

(A) Numbers of cells/well for cells plated on day 1 with 100,000 cells/well. Mean ± SD from 3 experiments.

(B) In normal cells, the metaphase plate aligns perpendicular to the substratum, with the mitotic spindle parallel to it.

(C) Spindle angles measured in fixed WT and HEK-IP$_3$R-KO cells using γ-tubulin staining to identify centrosomes. Individual values mean ± SD from 3 experiments. $^{***}P < 0.001$, Student's *t*-test.

(D) Brightfield images show that both daughter cells remain attached to the substratum during anaphase of a WT cell, while in the HEK-IP$_3$R-KO cell only one daughter cell remains attached. Scale bars = 20 μm

(E) Image of a multinucleate HEK-IP$_3$R-KO cell stained with α-tubulin (red) and DAPI (blue). Scale bar = 20 μm.

(F) Summary shows numbers of multinucleate cells. Mean ± SEM, *n* = 8 experiments, with 10 cells analysed in each. $^{*}P < 0.05$, Student's *t*-test.

(G) Typical example of a multipolar spindle in a HEK-IP$_3$R-KO cell stained for α-tubulin (red), γ-tubulin (yellow) and DNA (blue). Scale bar = 10 μm.

(H) Summary shows numbers of cells with multipolar spindles. Mean ± SEM, *n* = 3 dishes with 11 fields quantified in each. $^{**}P < 0.01$, Student's *t*-test.

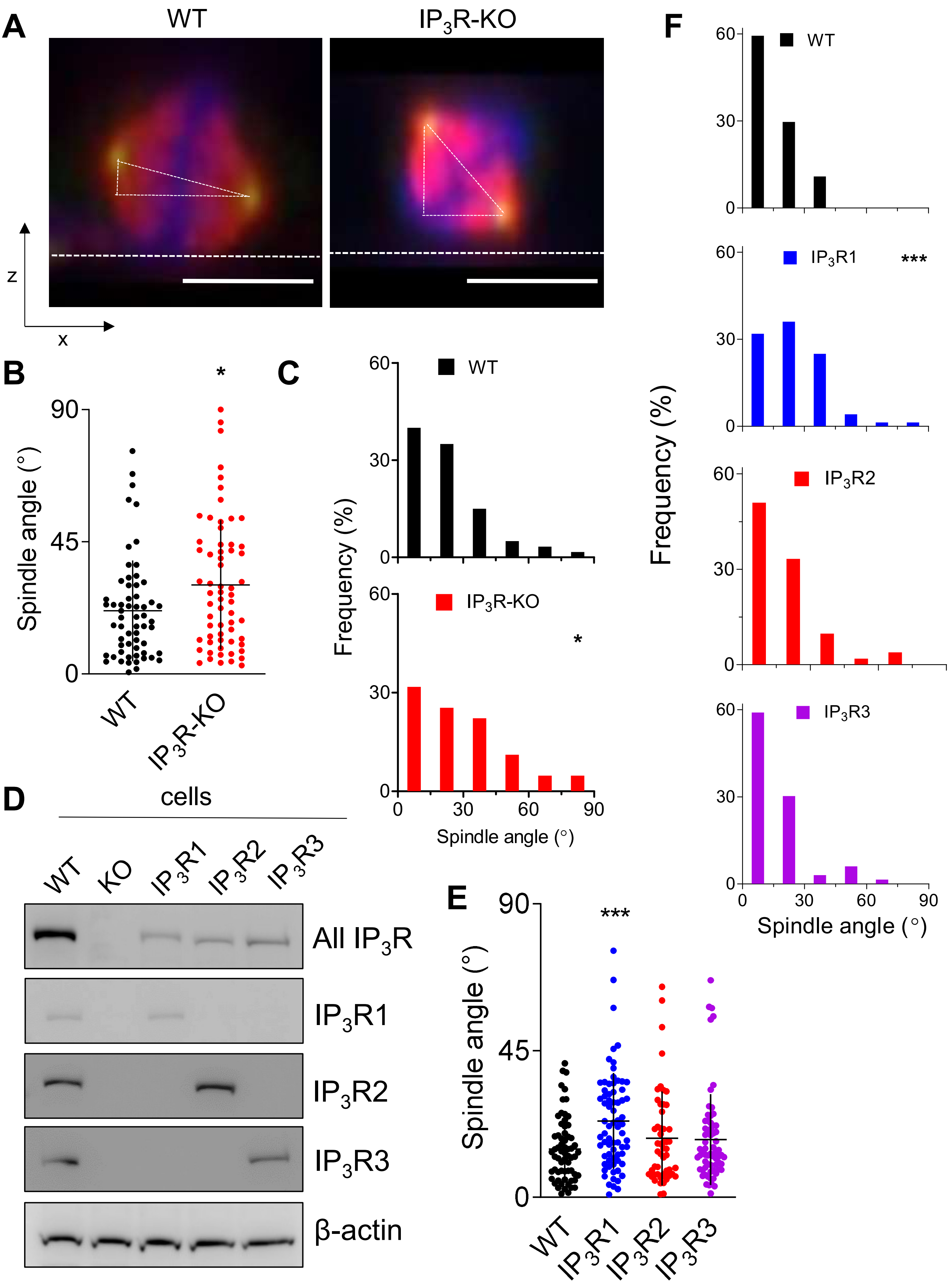

Figure S2. Spindle Angles in HAP1 Cells Without IP_3Rs and in HEK Cells Expressing Single IP_3R Subtypes. Related to Figure 1.

Legend on next page.

Figure S2. Spindle Angles in HAP1 Cells Without IP_3Rs and in HEK Cells Expressing Single IP_3R Subtypes. Related to Figure 1.
Figure on preceding page.

(A) Confocal z-stack images from fixed HAP1 cells show spindle angles during metaphase for a WT and HAP1-IP_3R-KO cell. The cells are immunostained for α-tubulin (red) and γ-tubulin (green), and stained with DAPI (blue) for chromosomes. Scale bars = 10 μm.
(B) Spindle angles for WT and HAP1-IP_3R-KO cells. Individual values, mean ± SD from 5 experiments. $^{*}P < 0.05$, Student's *t*-test.
(C) Frequency distribution of the spindle angles (data from panel B). $^{*}P < 0.05$, relative to WT, χ^2 test for trend.
(D) Typical western blots, using antibodies selective for IP_3R subtypes (IP_3R1-3) or an antibody that recognizes all three IP_3R subtypes (all IP_3R), show expression of IP_3R subtypes in WT HEK cells, HEK-IP_3R-KO cells and cells expressing single native IP_3R subtypes (Alzayady et al., 2016). The results replicate our previous analyses, which established that the HEK cell line used expresses 36% IP_3R1, 18% IP_3R2 and 45% IP_3R3 (Mataragka and Taylor, 2018).
(E) Spindle angles of cells expressing single IP_3R subtypes. Results show individual values, mean ± SD from 5 experiments. $^{***}P < 0.001$ relative to WT, ANOVA with Bonferroni test.
(F) Frequency distribution of spindle angles (data from panel B). $^{***}P < 0.001$, relative to WT, χ^2 test for trend.

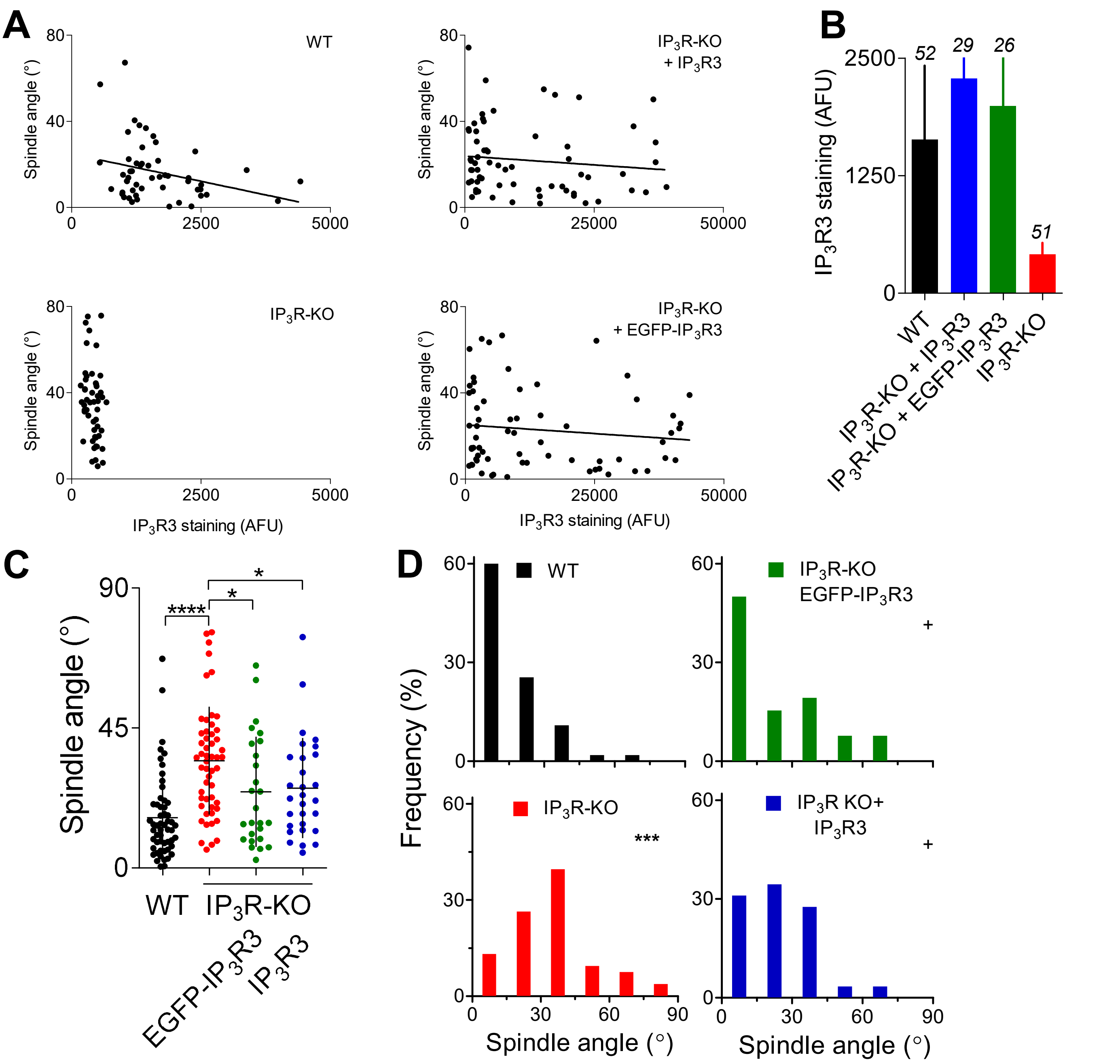

Figure S3. IP_3R3 and EGFP-IP_3R3 Similarly Rescue Spindle Alignment. Related to Figure 1.
(A) Relationships between immunostaining for IP_3R3 and spindle angle measured under identical conditions for WT and HEK-IP_3R-KO cells, or the latter expressing IP_3R3 or EGFP-IP_3R3. Points are from individual cells taken from 5 experiments. AFU, arbitrary fluorescence units (note the ten-fold difference in scale for WT and IP_3R-KO cells relative to transfected cells). For IP_3R-KO cells, all cells contributed to the analysis; whereas for the other cells only those with detectable immunostaining were included. From least-squares linear regression, the only slope to differ significantly from 0 was for WT cells ($P < 0.0001$). The results suggest firstly that the level of IP_3R expression in WT cells may contribute to whether the spindle aligns appropriately, and secondly that even massive over-expression of IP_3R does not perturb spindle alignment. Summary results in **Figures 1I and 1J**.
(B) Cells heterologously expressing IP_3R3 were selected (by immunostaining) to approximately match WT in their expression of IP_3R3. Note that in WT cells IP_3R3 comprises only ~45% of the IP_3Rs expressed (Mataragka and Taylor, 2018), whereas IP_3R3 is the only subtype in heterologously-expressing cells. Results show relative expression levels of IP_3R3 for cells used in the analyses shown in panels C and D.
(C) Spindle angles measured in the indicated cells, approximately matched for their expression of IP_3R3 (see panel B). Individual values, mean ± SD. $^{****}P < 0.0001$, $^{*}P < 0.05$, one-way ANOVA with Holm-Sidak multiple comparisons test, relative to HEK-IP_3R-KO cells.
(D) Distributions of spindle angles (data from panel C). $^{***}P < 0.001$, relative to WT, $^{+}P < 0.05$ relative to IP_3R-KO, χ^2 test for trend. Results shown here comprise a subset of the data (cells with IP_3R3 expression approximately matching expression in WT cells) shown in **Figures 1I and 1J**.

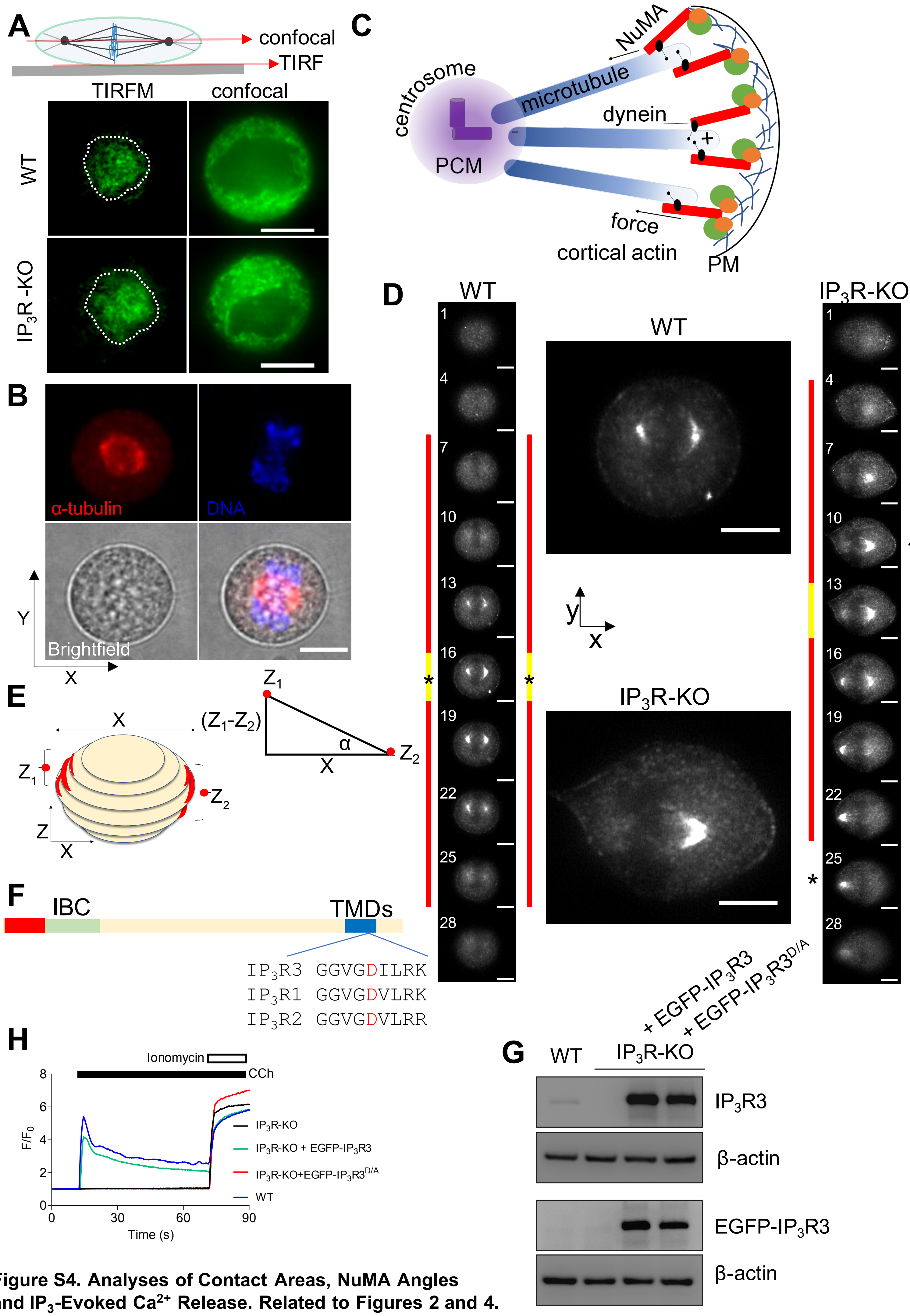

Figure S4. Analyses of Contact Areas, NuMA Angles and IP$_3$-Evoked Ca^{2+} Release. Related to Figures 2 and 4. Legend on next page.

Figure S4. Analyses of Contact Areas, NuMA Angles and IP_3-Evoked Ca^{2+} Release. Related to Figures 2 and 4.
Figure on preceding page.

(A) Confocal and TIRF microscopy was used to measure contact areas with the substratum for dividing cells with membranes labelled using DHCC (green). Scale bars = 10 µm. Summary results in **Figure 2A**.
(B) Typical confocal sections and brightfield images of isolated synchronized WT HEK cell expressing mCherry-α-tubulin and with DNA stained with NucBlue. Scale bar = 10 µm. Similar images were used to provide z-stacks from which spindle angles were measured (**Figures 2B-D**).
(C) NuMA links dynein to microtubules, and, through associated proteins, it associates with cortical actin. PCM, pericentriolar material.
(D) Examples of WT and HEK-IP_3R-KO cells in metaphase immunostained for NuMA. The large images show a single confocal section that includes a centrosome. Small images are sections (numbered from base of cell) showing locations of centrosomes (*) and the bounds of each NuMA crescent (red bars) and its centre (yellow bars, from which NuMA angles were calculated). NuMA angles were measured between the central points of the NuMA crescents relative to the substratum (**Figures 2E and 2F**). Scale bars = 10 µm.
(E) NuMA angles relative to the substratum were calculated as shown. The z-planes in which the centre of each NuMA crescent was located were identified (Z_1 and Z_2) and the angle between them (α) was calculated.
(F) Location of the mutated residue (D2477 of IP_3R3) within the selectivity filter that is conserved within all three IP_3R subtypes and falls within the re-entrant luminal loop linking transmembrane domains (TMD) 5 and 6. IBC, IP_3-binding core.
(G) Typical western blot (20 µg protein/lane) using antibodies to IP_3R3 (top) or GFP (bottom) of WT HEK cells or HEK-IP_3R-KO cells expressing the indicated IP_3R3 proteins. In 4 independent paired analyses, the intensity of the IP_3R3 band (determined using an Ab for IP_3R3) for cells expressing EGFP-$IP_3R3^{D/A}$ was 81.2 ± 4.5% that from cells expressing EGFP-IP_3R3.
(H) Ca^{2+} signals recorded using Calbryte-590 from HEK cells transiently expressing EGFP-IP_3R3s. Carbachol (CCh, 100 µM) was added to stimulate IP_3 formation through endogenous muscarinic acetylcholine receptors. Ionomycin (100 µM) was added to allow saturation of the Ca^{2+}-indicator. Results (F/F_0, where F is fluorescence recorded at each time, and F_0 fluorescence recorded at t = 0) are means from at least 22 successfully transfected cells from 3 experiments. The results establish that $IP_3R3^{D/A}$ does not mediate IP_3-evoked Ca^{2+} release (Boehning et al., 2001; Dellis et al., 2008).

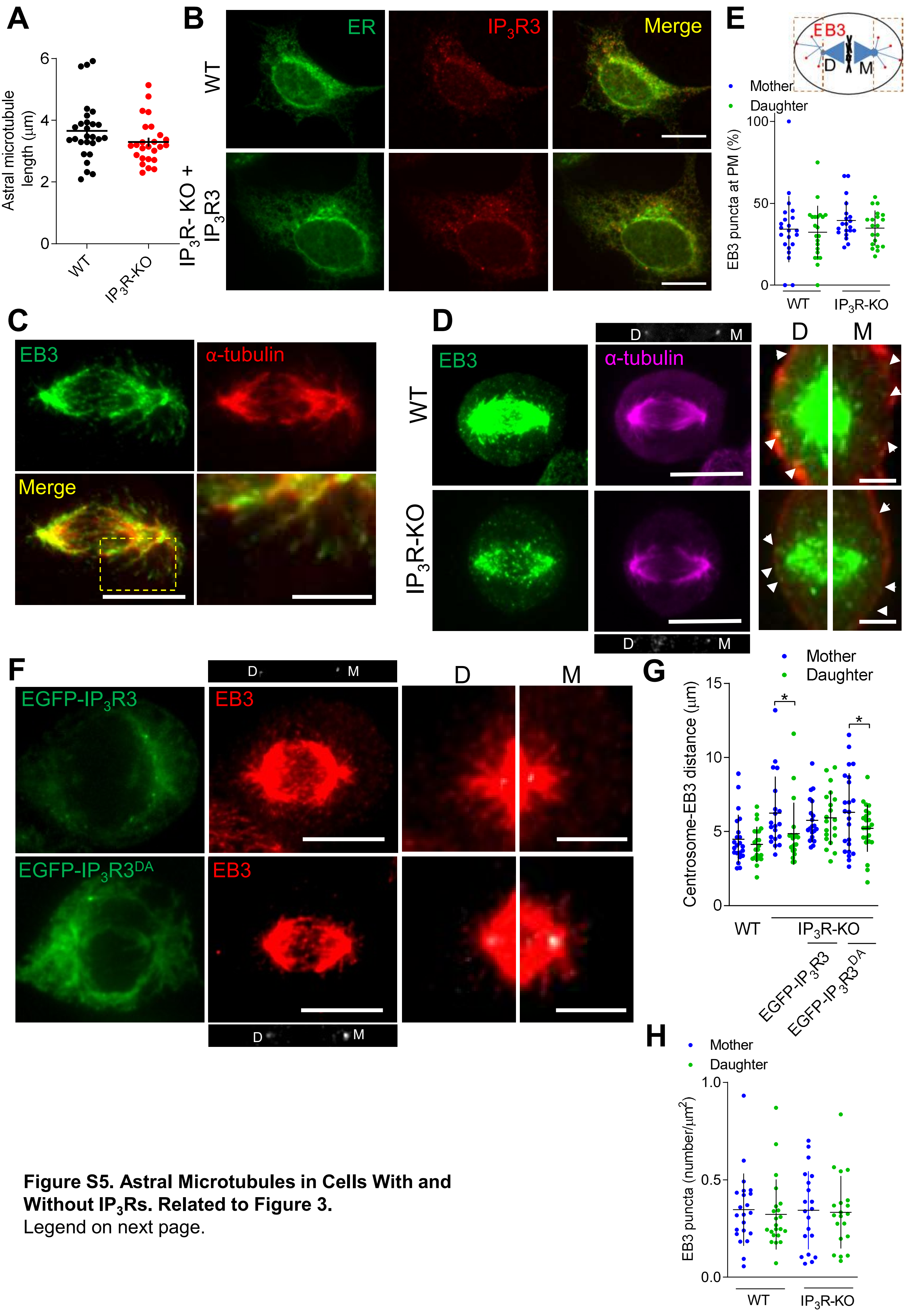

Figure S5. Astral Microtubules in Cells With and Without IP$_3$Rs. Related to Figure 3.
Legend on next page.

Figure S5. Astral Microtubules in Cells With and Without IP_3Rs. Related to Figure 3.
Figure on preceding page.

(A) Z-stacks from confocal sections that included both centrosomes (identified by cenexin staining) (**Figure 3C**) were used to measure lengths of astral microtubules at each pole of each cell (~8 measurements per cell). Results show mean values for each cell (without distinguishing mother and daughter centrosomes), and mean ± SEM for all cells. No significant difference between WT and HEK-IP_3R-KO cells, Student's *t*-test.
(B) Typical confocal images (central plane) show ER (GFP-ER, green) and immunostained IP_3R3 (red) in interphase WT HEK cells and HEK-IP_3R-KO cells expressing IP_3R3. Scale bars = 10 μm. Results confirm the similar subcellular distributions of native and heterologously expressed IP_3R3.
(C) Confocal image of HEK-IP_3R-KO cell immunostained for EB3 (green) and α-tubulin (red), showing EB3 capping astral microtubules. Scale bars = 10 μm (5 μm in enlargement of boxed area).
(D) Maximum intensity z-stack images around centrosome of HEK cells immunostained for EB3 (green), α-tubulin (magenta) and cenexin (grey), and with the plasma membrane (PM) identified using wheat germ agglutinin (WGA, red). Arrows show EB3 at the cell cortex (PM). Scale bars = 10 μm (5 μm in enlargements of areas surrounding centrosomes). D, daughter; M, mother.
(E) Summary results show the fraction of EB3 puncta located at the PM around mother and daughter centrosomes (areas shown in the inset) for cells with (WT, n = 22 cells) or without IP_3Rs (IP_3R-KO, n = 20 cells). Results show individual values and mean ± SD. No significant difference between any values, one-way ANOVA.
(F) Maximum intensity z-stack images around centrosome immunostained for EB3 (red) and cenexin (grey) in HEK-IP_3R-KO cells transfected with EGFP-IP_3R3 or EGFP-IP_3R3^{DA}. GFP-Booster was used to enhance the fluorescence of EGFP. Scale bars = 10 μm (5 μm in enlargements of regions surrounding centrosomes).
(G) Summary results show distance between centrosomes (cenexin) and EB3 in WT cells and HEK IP_3R-KO cells alone or after expression of EGFP-IP_3R3 or EGFP-IP_3R3^{DA}. Results show individual values, mean ± SD. $^{*}P < 0.05$, Paired student's *t*-test, mother relative to daughter centrosome.
(H) Numbers of immunostained EB3 puncta (#/μm^2) identified in regions around each centrosome (see inset to panel E). Results show individual values with mean ± SD from 22 (WT) or 20 (IP_3R-KO) cells. No significant differences, one-way ANOVA.
