# Supplementary material for: Ca2+ Release by IP3 Receptors Is Required to Orient the Mitotic Spindle
Source: Cell Rep. 2020 Dec 15;33(11):108483. doi: 10.1016/j.celrep.2020.108483 (PMC7758162; doi:10.1016/j.celrep.2020.108483)
Supplement: Document S2. Article plus Supplemental Information [file mmc4.pdf]

Cell Reports

Report

Ca^{2+} Release by IP_3 Receptors Is Required to Orient the Mitotic Spindle

Graphical Abstract

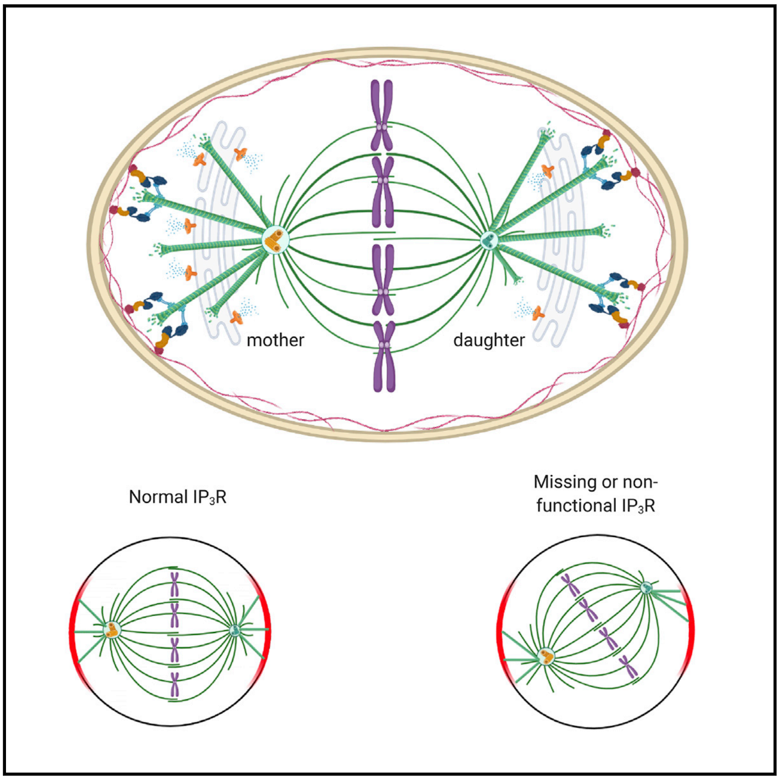

Highlights

- IP_3 receptors are required for mitotic spindle orientation
- Only IP_3 receptors with a functional channel restore spindle orientation
- Ca^{2+} release through IP_3 receptors is required for spindle orientation

Authors

Raul Lagos-Cabré, Adelina Ivanova, Colin W. Taylor

Correspondence

cwt1000@cam.ac.uk

In Brief

Lagos-Cabré et al. show that Ca^{2+} release by IP_3 receptors (IP_3Rs) regulates spindle orientation during metaphase. Spindles misalign in cells without IP_3Rs. Alignment is restored by expression of IP_3Rs but not by IP_3Rs that cannot release Ca^{2+}. Ca^{2+} release by IP_3Rs regulates astral microtubules and thereby spindle alignment.

Lagos-Cabré et al., 2020, Cell Reports *33*, 108483
December 15, 2020 © 2020 The Author(s).
https://doi.org/10.1016/j.celrep.2020.108483

CellPress

Cell Reports

CellPress
OPEN ACCESS

Report

Ca^{2+} Release by IP_3 Receptors Is Required to Orient the Mitotic Spindle

Raul Lagos-Cabré,[1] Adelina Ivanova,[1] and Colin W. Taylor[1,2,*]
[1]Department of Pharmacology, University of Cambridge, Tennis Court Road, Cambridge CB2 1PD, UK
[2]Lead Contact
*Correspondence: cwt1000@cam.ac.uk
https://doi.org/10.1016/j.celrep.2020.108483

SUMMARY

The mitotic spindle distributes chromosomes evenly to daughter cells during mitosis. The orientation of the spindle, guided by internal and external cues, determines the axis of cell division and thereby contributes to tissue morphogenesis. Progression through mitosis requires local Ca^{2+} signals at critical steps, and because store-operated Ca^{2+} entry is inhibited during mitosis, those signals probably require Ca^{2+} release through inositol 1,4,5-trisphosphate receptors (IP_3Rs). In cells without IP_3Rs, astral microtubules around the daughter centrosome are shorter than those at the mother centrosome, and the mitotic spindle fails to align with the substratum during metaphase. The misalignment is due to the spindle ineffectively detecting internal cues rather than a failure of cells to recognize the substratum. Expression of type 3 IP_3R is sufficient to rescue spindle alignment, but only if the IP_3R has a functional pore. We conclude that Ca^{2+} signals evoked by IP_3Rs are required to orient the mitotic spindle.

INTRODUCTION

During mitosis, centrosomes nucleate microtubules to form the mitotic spindle, which then distributes chromatids equally to daughter cells. The orientation of the spindle is important because it determines the plane of cell division and which of the daughter cells will receive the oldest (mother) centrosome (Bergstralh et al., 2017; di Pietro et al., 2016). Some stem cells, for example, selectively inherit the mother centrosome (Pelletier and Yamashita, 2012). For symmetric cell divisions, spindle alignment ensures that cellular components are shared equally between daughter cells, whereas for asymmetric divisions, spindle orientation determines whether one or both cells remain attached to the basement membrane (Hehnly et al., 2015; Lagos-Cabré and Moreno, 2008). Hence, spindle orientation ensures effective mitosis; it determines cell fate and which cells remain stem cells (Pelletier and Yamashita, 2012). Aberrant spindle alignment is associated with defective morphogenesis (Hehnly et al., 2015; Seldin et al., 2016), delamination of epithelia (Nakajima et al., 2013), and cancer (Bergstralh et al., 2017). Spindle positioning is achieved by interaction of astral microtubules emanating from the centrosomes with a protein complex anchored to the cell cortex. In vertebrates, this ternary complex includes nuclear mitotic apparatus (NuMA), LGN (for leucine-glycine-asparagine motifs), and the G-protein $G\alpha_i$ (Bergstralh et al., 2017). How cells regulate the interactions of astral microtubules with the cell cortex to ensure effective orientation of the mitotic spindle is not fully understood (di Pietro et al., 2016).

Ca^{2+} signals are associated with many key steps during mitosis, notably, at nuclear envelope breakdown, the transition from metaphase to anaphase and during cytokinesis (Humeau et al., 2018; Poenie et al., 1985, 1986; Whitaker and Patel, 1990; Wong et al., 2005). Ca^{2+} signals typically arise from a combination of Ca^{2+} release from intracellular stores, predominantly within the endoplasmic reticulum (ER), and Ca^{2+} entry across the plasma membrane. Store-operated Ca^{2+} entry, where loss of Ca^{2+} from the ER triggers opening of Ca^{2+} channels in the plasma membrane, is the most widely expressed Ca^{2+} entry pathway, but it is completely inhibited during mitosis (Smyth et al., 2009; Yu et al., 2019). Hence, mitotic Ca^{2+} signals are likely to be due entirely to release of Ca^{2+} from intracellular stores (Ciapa et al., 1994), most likely mediated by opening of inositol 1,4,5-trisphosphate receptors (IP_3Rs), which are ubiquitously expressed intracellular Ca^{2+} channels (Rossi and Taylor, 2018). Vertebrates express three closely related IP_3R subunits (IP_3R1–3) that are differentially expressed and differ in both their sensitivity to IP_3 and in their modulation by other signals, but each assembles to form homo- or hetero-tetrameric channels that open after IP_3 binding (Alzayady et al., 2016). It is noteworthy that IP_3Rs are phosphorylated by several mitosis-related kinases, including polo-like kinase 1 (PLK1), cyclin-dependent kinase 1 (CDK1), and extracellular signal-regulated kinases 1 and 2 (ERK1/2), each of which regulates responses to IP_3 (Ito et al., 2008; Prole and Taylor, 2016; Sathanawongs et al., 2015). Furthermore, mitosis is accompanied by substantial subcellular redistribution of the ER and IP_3Rs (Mitsuyama and Sawai, 2001; Parry et al., 2005).

We show that in cells without IP_3Rs, the mitotic spindle fails to align properly. Although the NuMA crescent aligns appropriately in cells lacking IP_3Rs, the spindle fails to align with that internal cue and astral microtubules around the daughter centrosome are shorter than those emanating from the mother centrosome.

This is an open access article under the CC BY license (http://creativecommons.org/licenses/by/4.0/).

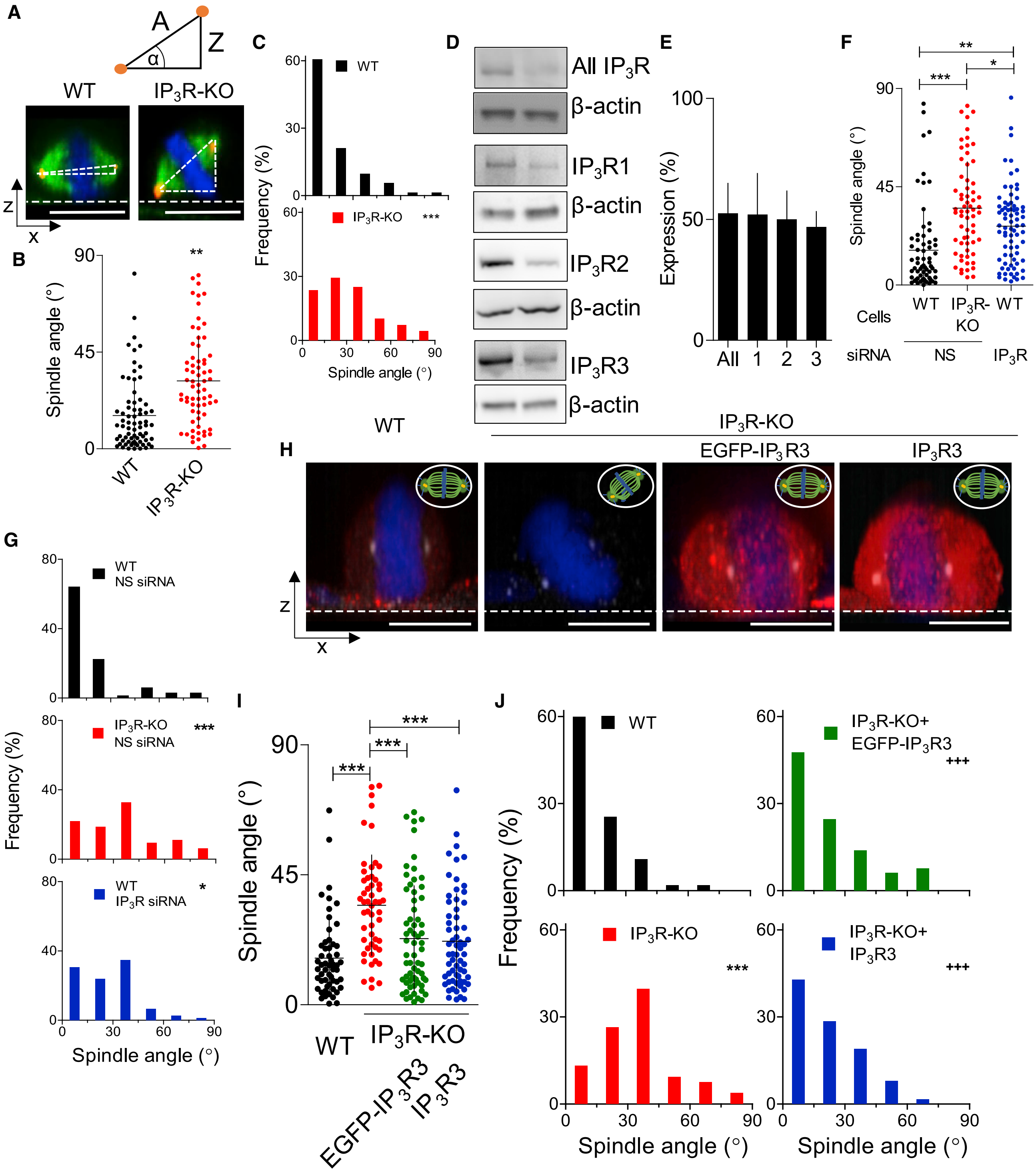

Figure 1. Loss of IP_3Rs Causes Misalignment of Mitotic Spindles

(A) Typical projections of confocal z stacks from cells stained for chromosomes (blue), α-tubulin (green), and γ-tubulin (red) show spindle angles (α) during metaphase for a WT and HEK-IP_3R-KO cell. Dashed lines show substratum. Scale bars: 10 μm.

(B) Spindle angles for WT and HEK-IP_3R-KO cells (individual values, means ± SD from five experiments). $^{**}p < 0.01$, Student's t test.

(C) Frequency distribution of spindle angles (n = 68–71 cells, from five experiments). $^{***}p < 0.001$, χ^2 test for trend.

(D) Typical western blots (WBs) for IP_3R subtypes in HEK cells treated with siRNAs to all three IP_3R subtypes or non-silencing (NS) siRNA.

(E) Summary results show IP_3R expression determined by quantification of WB for cells treated with IP_3R siRNA relative to NS siRNA (%, means ± SD, n = 4).

(legend continued on next page)

Expression of IP_3R3 rescues spindle alignment, but only if the IP_3R has a functional pore. We conclude that Ca^{2+} release by IP_3Rs, which accumulate around centrosomes during metaphase and more so at the mother centrosome, is required for spindle alignment.

RESULTS

IP_3 Receptors Are Required for the Mitotic Spindle to Align with the Substratum

Human embryonic kidney (HEK) cells without IP_3Rs (HEK-IP_3R-KO cells) and wild-type (WT) cells grew at similar rates (Figure S1A), confirming that IP_3Rs are not essential for proliferation (Ando et al., 2018; Atakpa et al., 2019; Sugawara et al., 1997). Time-lapse images of dividing cells showed that most WT cells formed a metaphase plate that aligned perpendicular to the substratum, allowing each daughter cell to remain attached to the substratum after cytokinesis (Figure S1B). This behavior is common to almost all epithelial cells and cell lines (Hehnly et al., 2015). However, many HEK-IP_3R-KO cells formed a metaphase plate that was not perpendicular to the substratum, allowing only one daughter cell to remain attached, whereas the other was expelled into the medium (Figures S1C and S1D; Video S1). Imaging of cells expressing mCherry-α-tubulin and histone-2B-EGFP, to identify microtubules and chromosomes, respectively, showed that mitotic spindles in WT cells remained parallel to the substratum throughout metaphase, whereas, in HEK-IP_3R-KO cells, the spindle rotated (Videos S2A and S2B). The misaligned spindles in HEK-IP_3R-KO cells were often associated with aberrant mitoses, including nuclear division without cytokinesis (Video S2C), consistent with many cells having several nuclei or multipolar spindles (Figures S1E–S1H). The results are also consistent with a small interfering RNA (siRNA) screen, where loss of IP_3R1 was associated with abnormal cytokinesis (Kittler et al., 2004). These observations prompted us to examine the relationship between IP_3Rs and the mitotic spindle.

We measured spindle angles relative to the substratum for semi-confluent HEK cells during metaphase (Figure 1A). In WT cells, most spindles aligned parallel to the substratum (a perpendicular metaphase plate), with an average spindle angle of 15.5° ± 16.4° (mean ± SD, n = 71 cells). Because the angles always have positive values, any variation causes the mean to deviate from the 0° that would indicate perfect alignment. In HEK-IP_3R-KO cells, the bipolar spindles were more randomly oriented, evident from both the larger average spindle angle (31.6° ± 21.0°, n = 68) and the wider distribution of the angles (Figures 1B and 1C). Similar results were obtained using γ-tubulin to define spindle angles (Figure S1C) and from analyses of HAP1 cells with and without IP_3Rs (Figures S2A–S2C).

Because the cell lines lacking IP_3Rs were generated by selection after CRISPR/Cas9-mediated gene disruption (Alzayady et al., 2016; Atakpa et al., 2018), the misaligned spindles might have arisen through effects unrelated to the loss of IP_3Rs. We, therefore, used siRNAs directed to each IP_3R subtype to acutely reduce expression of all three IP_3R subtypes in HEK cells. The siRNA treatment reduced expression of the IP_3R subtypes similarly (Figures 1D and 1E). Spindle angles were significantly perturbed in siRNA-treated cells (26.9° ± 17.5°, n = 75), although less so than in HEK-IP_3R-KO cells (35.2° ± 21.2°, n = 64) (Figure 1F). The distribution of spindle angles confirmed that loss of IP_3Rs by siRNA or gene disruption had similar effects (Figure 1G).

HEK cells express all three IP_3R subtypes (Mataragka and Taylor, 2018). In cells expressing only a single IP_3R subtype, spindle angles were aberrant in cells expressing only IP_3R1 (23.4° ± 14.7°, n = 72), but more normal in cells expressing only IP_3R2 (18.1° ± 14.4°, n = 51) or IP_3R3 (17.7° ± 13.9°, n = 66) (Figures S2D–S2F). We cannot conclude from these results that IP_3R1 is incapable of contributing to spindle alignment because the HEK-IP_3R1 cells may have acquired other defects during selection, but the results do establish that the requirement for IP_3Rs can be satisfied by IP_3R2 or IP_3R3. Our subsequent studies focus on IP_3R3 because plasmids encoding it are more reliably propagated than those expressing IP_3R2, and there is a better antibody to IP_3R3.

In HEK-IP_3R-KO cells, the spindle angle (34.4° ± 17.4°, n = 53) was significantly rescued by expression of EGFP-IP_3R3 (22.9° ± 18.3°, n = 65) to values that were not significantly different from the mock-transfected WT cells (16.2° ± 13.4°, n = 55) (Figures 1H–1J). We confirmed that the EGFP tag did not affect IP_3R3 function by demonstrating that, for matched levels of IP_3R3 expression (and comparable to WT cells), rescue of spindle alignment was indistinguishable for EGFP-IP_3R3 (22.9° ± 18.3°) and untagged IP_3R3 (22.0° ± 16.4°, n = 63 cells) (Figures 1H–1J and S3). Furthermore, expression of IP_3R3 at levels substantially exceeding native levels did not perturb spindle alignment (Figure S3A). In WT cells, however, endogenous IP_3R3 expression inversely correlated with spindle angle, suggesting that native levels of IP_3R expression may be limiting for appropriate spindle alignment (Figure S3A). The results so far establish that IP_3Rs are required for the mitotic spindle to align

(F) Spindle angles for matched comparisons of WT and HEK-IP_3R-KO cells and cells treated with NS or IP_3R siRNA (individual values, means ± SD from five experiments). *p < 0.05, **p < 0.01, ***p < 0.001, ANOVA with Bonferroni test.

(G) Frequency distributions of spindle angles for cells (WT or IP_3R-KO) treated with NS or IP_3R siRNA (n = 64–75 cells, from five experiments). ***p < 0.001, *p < 0.05, relative to WT with NS siRNA, χ^2 test for trend.

(H) Cross-sections through 3D reconstructions of confocal z stack images of mitotic HEK cells expressing EGFP-IP_3R3 or untagged IP_3R3, immunostained for IP_3R3 (red) and showing chromosomes (DAPI, blue) and γ-tubulin (white). Scale bars: 10 μm.

(I) Spindle angles for WT and HEK-IP_3R-KO cells transiently expressing IP_3R3 or EGFP-IP_3R3. Results (individual values, means ± SD, from five experiments). *p < 0.05, **p < 0.01, ANOVA with Bonferroni test.

(J) Frequency distributions of spindle angles (n = 53–65 cells, from five experiments). ***p < 0.001, relative to WT; $^{+++}$p < 0.001, relative to IP_3R-KO, χ^2 test for trend.

See also Figures S1–S3 and Videos S1 and S2.

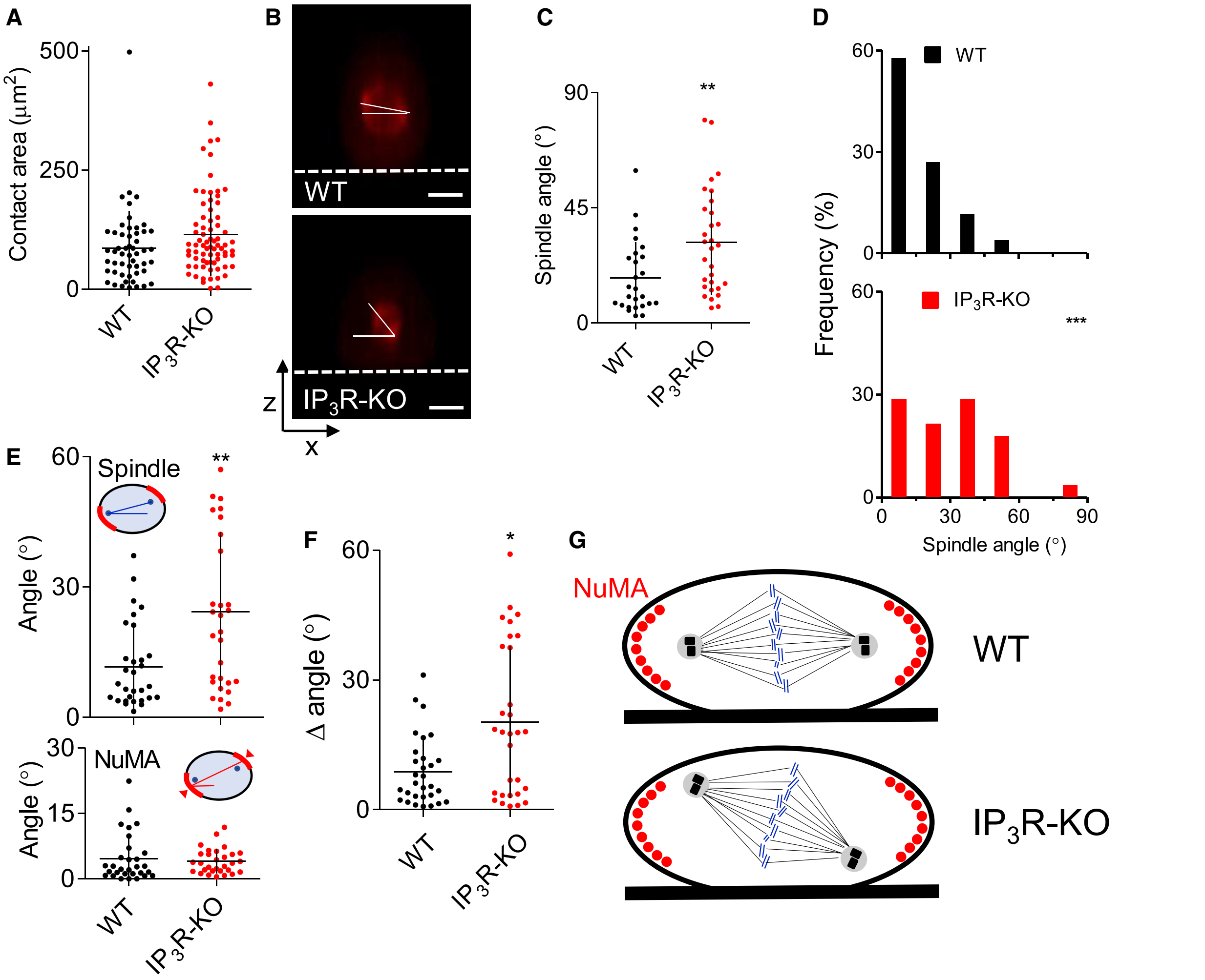

Figure 2. IP_3Rs Are Required for Spindles to Align with Intracellular Cues

(A) Contact areas between PM and substratum (individual values from five experiments, means ± SD). $p > 0.05$, Student's t test. From Figure S4A.

(B) Side view of confocal z stacks showing spindle angles identified by mCherry-α-tubulin in isolated cells. Dashed lines show substratum. Scale bars: 10 μm.

(C) Spindle angles in isolated synchronized HEK cells with no intercellular contacts, from (B). Individual values, means ± SD from three experiments. **$p < 0.01$, Student's t test.

(D) Frequency distribution of spindle angles in isolated cells (n = 26–28 cells, from three experiments). ***$p < 0.001$, χ^2 test for trend.

(E) Spindle (from γ-tubulin staining, top) and NuMA angles (bottom) in WT and HEK-IP_3R-KO cells, each measured relative to the substratum. Individual values, means ± SD from four experiments. **$p < 0.01$, Student's t test.

(F) Difference (Δ) between spindle and NuMA angles for individual cells. Individual values, means ± SD, n = 4 experiments. *$p < 0.05$, Student's t test.

(G) NuMA aligns normally in IP_3R-KO cells, but centrosomes no longer align appropriately with NuMA.

See also Figures S4A–S4E.

with the substratum and that IP_3R3 (or EGFP-IP_3R3) is sufficient to meet that need.

IP_3 Receptors Are Required to Align Spindles with the NuMA Complex

Spindle angles were measured relative to the substratum (Figures 1A and S1B) using semi-confluent cells, in which most cells contact at least one neighbor. We therefore considered whether spindle misalignment in cells without IP_3Rs arose from ineffective decoding of appropriate intracellular signals by the spindle apparatus or from ineffective detection of external cues, for example β1-integrin-mediated interaction with the extracellular matrix (Iwano et al., 2015) or cadherin-mediated interactions between cells (Hart et al., 2017).

Using synchronized dividing cells stained with a lipid marker, we confirmed that HEK-IP_3R-KO cells adhere normally and expose similar areas of plasma membrane (PM) to the substratum as WT cells (Figures 2A and S4A). In sparsely distributed synchronized WT cells expressing mCherry-α-tubulin, spindles aligned with the substratum (17.6° ± 14.1°, n = 26) (Figures 2B and S4B), consistent with published results from isolated HeLa cells (Toyoshima et al., 2007), but again, the spindles were misaligned in HEK-IP_3R-KO cells (31.4° ± 20.3°, n = 30) (Figures 2B–2D). We conclude that

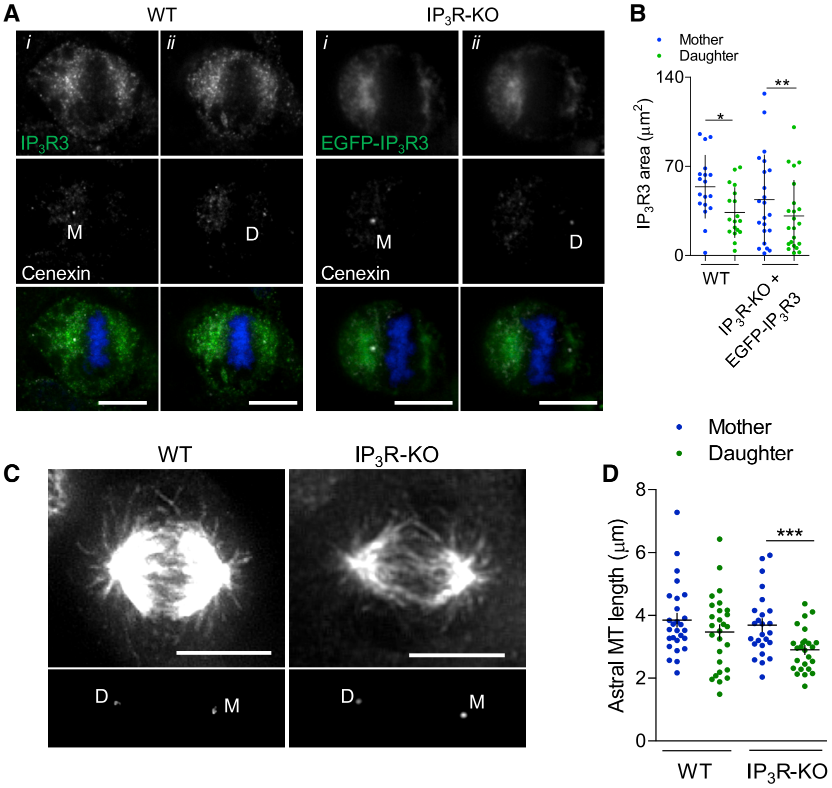

Figure 3. IP_3R-Evoked Ca^{2+} Signals Are Required for Spindle Alignment

(A) Confocal images of metaphase WT cells or HEK-IP_3R-KO cells expressing EGFP-IP_3R3, immunostained for IP_3R3 (for WT) and cenexin (white, to distinguish mother [M] and daughter [D] centrosomes). Images show sections in which the mother (*i*) or daughter (*ii*) centrosome was most intensely stained. Overlay images show IP_3R (green, EGFP or immunostaining), cenexin (white), and DNA (DAPI, blue). Scale bars: 10 μm.

(B) Summary results (individual values, means ± SD, from three experiments) show areas populated by IP_3R3 surrounding the two centrosomes (immunostaining or EGFF). *$p < 0.05$, **$p < 0.01$, paired Student's t test for mother relative to daughter.

(C) Z stacks of five confocal sections show astral microtubules (α-tubulin staining) and mother (M) and daughter (D) centrosomes (cenexin staining). Scale bars: 10 μm.

(D) Summary results (individual values, means ± SEM) show mean lengths of astral microtubules in individual cells associated with mother and daughter centrosomes (typically four measurements for each centrosome in each cell). ***$p < 0.001$, Student's t test.

See also Figure S5.

misaligned spindles in cells without IP_3Rs are not due to defects in either attachment to the substratum or communication between cells.

In vertebrates, NuMA is released from the nucleus during nuclear envelope breakdown. NuMA, associated with the microtubule motor dynein, then forms a complex at the PM to orient the spindle poles (Bergstralh et al., 2017; Du and Macara, 2004; Gloerich et al., 2017) (Figure S4C). NuMA expression (99.0% ± 9.9%, n = 3, determined by western blot [WB]) and the lengths of NuMA crescents (19.3 ± 8.0 μm and 21.0 ± 7.2 μm) were indistinguishable in WT and IP_3R-KO cells. We investigated whether the need for IP_3Rs arose upstream or downstream of NuMA complexes by measuring spindle angles and angles between the centers of NuMA crescents in the same metaphase cells. Spindles were misaligned in HEK-IP_3R-KO cells (24.3° ± 18.4° versus 11.6° ± 9.6° in WT cells, n > 30), but the NuMA angles were indistinguishable in cells with (4.6° ± 5.5°, n = 30) and without (4.0° ± 2.8°, n = 30) IP_3Rs (Figures 2E, S4D, and S4E). The disjunction of NuMA and the spindle in cells without IP_3Rs is clear from comparison of the difference in their angles within individual cells (Δ = 8.23° ± 7.96° and 20.2° ± 17.5° in WT and IP_3R-KO cells, respectively, n = 30) (Figure 2F). These results suggest that IP_3Rs are not required to align NuMA with the substratum, but they are required for the spindle to align with the NuMA complex (Figure 2G).

Loss of IP_3Rs had no significant effect on the mean length of the astral microtubules measured in z stacks of confocal images (Figure S5A). The mother centrosome, which contains the oldest centrosome, more effectively nucleates microtubules, forms more extensive astral microtubules during mitosis, contributes to cell polarity, and can affect cell fate (Hehnly et al., 2015; Yamashita, 2009). In WT HEK cells and in HEK-IP_3R-KO cells expressing EGFP-IP_3R3 (wherein the distribution of IP_3R3 mimics that of WT cells; Figure S5B), IP_3R3 concentrated most around the mother centrosome during metaphase (Figures 3A and 3B). Indistinguishable results were obtained using immunostaining (WT cells) and EGFP-fluorescence (HEK-IP_3R-KO cells expressing EGFP-IP_3R3) (Figure 3B). These observations prompted us to determine separately the length of astral microtubules around mother and daughter centrosomes. The results indicate that in HEK-IP_3R-KO cells, the astral microtubules around the daughter centrosome are shorter than those around the mother centrosome (Figures 3C, 3D, S5C, S5D, S5F, and S5G). There were no significant differences between cells with and without IP_3Rs in the length of the mitotic spindle (9.44 ± 1.27 μm, n = 53 in WT cells, and 9.99 ± 1.41 μm, n = 57 in HEK-IP_3R-KO cells, mean ± SD), the distance between the cortex and mother centrosome (5.36 ± 2.35 μm, n = 26 and 4.86 ± 2.85 μm, n = 25) or daughter centrosome (5.81 ± 2.57 μm and 4.55 ± 2.02 μm), the fraction of the plus ends of microtubules (identified with end-binding protein 3, EB3) abutting the cortex around the mother or daughter centrosome (Figures S5C–S5E), or the density of EB3 puncta around the centrosomes (Figure S5H)

Collectively, these results establish that IP_3Rs are required for centrosomes to align appropriately with internal cues provided

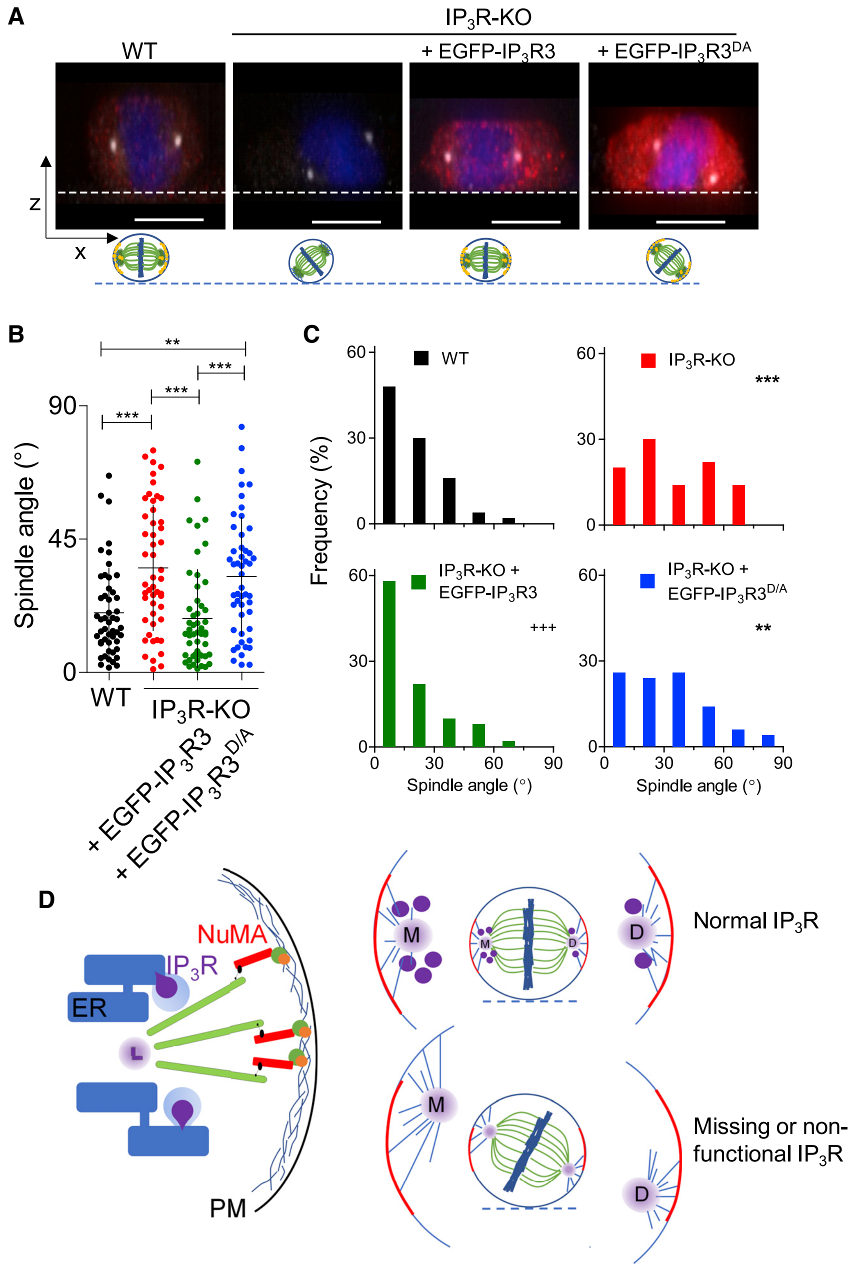

Figure 4. Ca^{2+} Signals Evoked by IP_3Rs Are Required for Spindle Alignment

(A) Side views of 3D-projected z stacks from metaphase cells immunostained for IP_3R3 (red) and γ-tubulin (gray) and with DNA stained with DAPI (blue). Dashed lines show substratum. Scale bar: 10 μm.

(B) Spindle angles (individual values, means ± SD, n = 50 cells from five experiments). $^{***}p < 0.001$, $^{**}p < 0.01$, ANOVA with Bonferroni test.

(C) Frequency distribution of spindle angles (n = 50 cells from five experiments for each condition). $^{***}p < 0.001$, $^{**}p < 0.01$, relative to WT; $^{+++}p < 0.001$, relative to IP_3R-KO, χ^2 test for trend.

(D) During metaphase, IP_3Rs (purple circles) accumulate around the centrosomes, more so around the mother (M) than the daughter (D) centrosome. Astral microtubules, through their association with NuMA and cortical actin, control orientation of the mitotic spindle. IP_3R-evoked Ca^{2+} signals, by regulating astral microtubules, influence spindle orientation. In cells without functional IP_3Rs, astral microtubules around the mother centrosome are longer and the spindles less effectively align with NuMA.

See also Figures S4F–S4H.

by the NuMA complex and that astral microtubules radiating from mother and daughter centrosomes are differentially affected by the loss of IP_3Rs.

Ca^{2+} Release by IP_3 Receptors Is Required for Spindle Alignment

IP_3Rs are best known for releasing Ca^{2+} from the ER (Rossi and Taylor, 2018), but additional proteins associate with IP_3Rs (Prole and Taylor, 2016), and many IP_3Rs appear not to release Ca^{2+} in intact cells (Thillaiappan et al., 2017). We, therefore, considered whether Ca^{2+} release through IP_3Rs was required for spindle alignment. Mutation of a single residue within the pore of IP_3R1 prevents it from conducting Ca^{2+} (Boehning et al., 2001; Dellis et al., 2008). We mutated the equivalent residue (D2477A) in EGFP-IP_3R3 (EGFP-$IP_3R3^{D/A}$) and confirmed that it prevented IP_3 from evoking Ca^{2+} release (Figures S4F–S4H). Expression of EGFP-IP_3R3 in HEK IP_3R-KO cells rescued spindle alignment, and the similar lengths of astral microtubules at mother and daughter centrosomes were restored, but comparable expression of EGFP-$IP_3R3^{D/A}$ rescued neither feature (Figures 4A–4C, S5F, and S5G). These results establish that Ca^{2+} release through IP_3Rs is required for mitotic spindles to align properly (Figure 4D).

DISCUSSION

Progression through the cell cycle is controlled by regulated degradation of cyclins, but local Ca^{2+} signals are also important at critical stages (Keith et al., 1985; Parry et al., 2005; Zhao et al., 2019), including nuclear envelope breakdown, the transition from metaphase to anaphase and cytokinesis. The mechanisms are largely unresolved, but because store-operated Ca^{2+} entry is inhibited during meiosis and mitosis (Smyth and Putney, 2012; Yu et al., 2019), the Ca^{2+} signals are probably evoked by IP_3Rs. We have shown that cells can proliferate without IP_3Rs (Figure S1A) (Alzayady et al., 2016; Ando et al., 2018; Atakpa et al., 2019; Sugawara et al., 1997), but their behavior is compromised. In some cells without IP_3Rs, the mitotic spindle is aberrant and cytokinesis sometimes fails (Figure S1). However, most cells divide

successfully but have spindles that fail to align with the substratum because they no longer associate appropriately with the NuMA complex (Figures 1 and 2). The defects are reversed by expression of IP_3R3, but only if it has a functional Ca^{2+} pore (Figure 4). This is consistent with recent work suggesting that local Ca^{2+} signals occur near centrosomes throughout mitosis, and abolishing them at one centrosome perturbs mitosis (Helassa et al., 2019). It is noteworthy that variations in expression of endogenous IP_3Rs are associated with the reliability of spindle alignment: WT cells with fewer IP_3Rs are more likely to have misaligned spindles (Figure S3A).

Astral microtubules link the NuMA complex to the pericentriolar material (PCM) that surrounds each centrosome (Figure S4C). IP_3Rs concentrate most around the mother centrosome (Figures 3A and 3B), which nucleates astral microtubules that are more abundant and longer than those associated with the daughter centrosome (Hehnly et al., 2015; Yamashita, 2009). There is a significant disparity in the length of astral microtubules emanating from the mother and daughter centrosomes in cells without IP_3Rs: astral microtubules at the mother pole are longer than those at the daughter pole (Figures 3C, 3D, S5F, and S5G), although they appear to make similar numbers of contacts with the cortex at both poles in cells with or without IP_3Rs (Figures S5C–S5E). Our results indicate an asymmetric relationship between IP_3Rs and the mother and daughter centrosomes. The mother centrosome has greater capacity to nucleate microtubules and associates with additional proteins (Huhn et al., 2017) and IP_3Rs concentrate most around it (Figures 3A and 3B), and in cells without IP_3Rs, there is a relative lengthening of astral microtubules around the mother centrosome (Figures 3C, 3D, S5F, and S5G). We speculate that the asymmetric lengths of astral microtubules around mother and daughter centrosomes in cells without IP_3Rs may generate uneven forces between them and the cortex (Howard and Garzon-Coral, 2017), leading to spindle misalignment.

We conclude that Ca^{2+} release through IP_3Rs is required for the mitotic spindle to align appropriately, probably through regulation of astral microtubules by IP_3R-evoked Ca^{2+} signals (Figure 4D). Future experiments should assess whether the Ca^{2+} signals that occur during metaphase (Helassa et al., 2019) regulate microtubules and their motors or deliver Ca^{2+} locally to the mitochondria to allow local delivery of ATP (Zhao et al., 2019) and thereby regulation of microtubule activity. Our results reveal that IP_3R-evoked Ca^{2+} signals are an important means of regulating the mitotic spindle.

STAR★METHODS

Detailed methods are provided in the online version of this paper and include the following:

- KEY RESOURCES TABLE
- RESOURCE AVAILABILITY
  - Lead Contact
  - Materials Availability
  - Data and Code Availability
- EXPERIMENTAL MODEL AND SUBJECT DETAILS
- METHOD DETAILS
  - Cell Culture and Transfection
  - Immunocytochemistry
  - Live-Cell Imaging
  - Measurement of Contact Areas Between Cells and Substratum
  - Measurements of Cytosolic Ca^{2+} Signals
  - Western Blots
- QUANTIFICATION AND STATISTICAL ANALYSIS
  - Measurements of Angles of Mitotic Spindles and NuMA Crescents
  - Measurements of IP_3R Distributions Around Mother and Daughter Centrosomes
  - Measurements of Lengths of Astral Microtubules
  - Statistical Analyses

SUPPLEMENTAL INFORMATION

Supplemental Information can be found online at https://doi.org/10.1016/j.celrep.2020.108483.

ACKNOWLEDGMENTS

This work was supported by Wellcome (101844) and the Biotechnology and Biological Sciences Research Council (BB/S013776/1). We thank Dr. David Yule (University of Rochester, Rochester, NY, USA) for making HEK cell lines expressing no IP_3R or single IP_3R subtypes available.

AUTHOR CONTRIBUTIONS

R.L.-C. performed most experiments. A.I. analyzed IP_3R localization. C.W.T. supervised the study and wrote the manuscript. All authors contributed to data analysis and reviewed the manuscript.

DECLARATION OF INTERESTS

The authors declare no competing interests.

Received: January 7, 2020
Revised: September 23, 2020
Accepted: November 13, 2020
Published: December 15, 2020

REFERENCES

Alzayady, K.J., Wang, L., Chandrasekhar, R., Wagner, L.E., 2nd, Van Petegem, F., and Yule, D.I. (2016). Defining the stoichiometry of inositol 1,4,5-trisphosphate binding required to initiate Ca^{2+} release. Sci. Signal. *9*, ra35.

Ando, H., Hirose, M., and Mikoshiba, K. (2018). Aberrant IP_3 receptor activities revealed by comprehensive analysis of pathological mutations causing spinocerebellar ataxia 29. Proc. Natl. Acad. Sci. USA *115*, 12259–12264.

Atakpa, P., Thillaiappan, N.B., Mataragka, S., Prole, D.L., and Taylor, C.W. (2018). IP_3 receptors preferentially associate with ER-lysosome contact sites and selectively deliver Ca^{2+} to lysosomes. Cell Rep. *25*, 3180–3193.e7.

Atakpa, P., van Marrewijk, L.M., Apta-Smith, M., Chakraborty, S., and Taylor, C.W. (2019). GPN does not release lysosomal Ca^{2+} but evokes Ca^{2+} release from the ER by increasing the cytosolic pH independently of cathepsin C. J. Cell Sci. *132*, jcs223883.

Bergstralh, D.T., Dawney, N.S., and St Johnston, D. (2017). Spindle orientation: a question of complex positioning. Development *144*, 1137–1145.

Boehning, D., Mak, D.-O., Foskett, J.K., and Joseph, S.K. (2001). Molecular determinants of ion permeation and selectivity in inositol 1,4,5-trisphosphate receptor Ca^{2+} channels. J. Biol. Chem. *276*, 13509–13512.

Ciapa, B., Pesando, D., Wilding, M., and Whitaker, M. (1994). Cell-cycle calcium transients driven by cyclic changes in inositol trisphosphate levels. Nature *368*, 875–878.

Colicino, E.G., Stevens, K., Curtis, E., Rathbun, L., Manikas, J., Amack, J., Freshour, J., and Hehnly, H. (2019). Chromosome misalignment is associated with PLK1 activity at cenexin-positive mitotic centrosomes. Mol. Biol. Cell *30*, 1598–1609.

Dellis, O., Rossi, A.M., Dedos, S.G., and Taylor, C.W. (2008). Counting functional IP_3 receptors into the plasma membrane. J. Biol. Chem. *283*, 751–755.

di Pietro, F., Echard, A., and Morin, X. (2016). Regulation of mitotic spindle orientation: an integrated view. EMBO Rep. *17*, 1106–1130.

Du, Q., and Macara, I.G. (2004). Mammalian Pins is a conformational switch that links NuMA to heterotrimeric G proteins. Cell *119*, 503–516.

Gloerich, M., Bianchini, J.M., Siemers, K.A., Cohen, D.J., and Nelson, W.J. (2017). Cell division orientation is coupled to cell-cell adhesion by the E-cadherin/LGN complex. Nat. Commun. *8*, 13996.

Hart, K.C., Tan, J., Siemers, K.A., Sim, J.Y., Pruitt, B.L., Nelson, W.J., and Gloerich, M. (2017). E-cadherin and LGN align epithelial cell divisions with tissue tension independently of cell shape. Proc. Natl. Acad. Sci. USA *114*, E5845–E5853.

Hehnly, H., Canton, D., Bucko, P., Langeberg, L.K., Ogier, L., Gelman, I., Santana, L.F., Wordeman, L., and Scott, J.D. (2015). A mitotic kinase scaffold depleted in testicular seminomas impacts spindle orientation in germ line stem cells. eLife *4*, e09384.

Helassa, N., Nugues, C., Rajamanoharan, D., Burgoyne, R.D., and Haynes, L.P. (2019). A centrosome-localized calcium signal is essential for mammalian cell mitosis. FASEB J. *33*, 14602–14610.

Howard, J., and Garzon-Coral, C. (2017). Physical limits on the precision of mitotic spindle positioning by microtubule pushing forces: mechanics of mitotic spindle positioning. BioEssays *39*, 1700122.

Huhn, S.C., Liu, J., Ye, C., Lu, H., Jiang, X., Feng, X., Ganesan, S., White, E., and Shen, Z. (2017). Regulation of spindle integrity and mitotic fidelity by BCCIP. Oncogene *36*, 4750–4766.

Humeau, J., Bravo-San Pedro, J.M., Vitale, I., Nuñez, L., Villalobos, C., Kroemer, G., and Senovilla, L. (2018). Calcium signaling and cell cycle: progression or death. Cell Calcium *70*, 3–15.

Ito, J., Yoon, S.Y., Lee, B., Vanderheyden, V., Vermassen, E., Wojcikiewicz, R., Alfandari, D., De Smedt, H., Parys, J.B., and Fissore, R.A. (2008). Inositol 1,4,5-trisphosphate receptor 1, a widespread Ca^{2+} channel, is a novel substrate of polo-like kinase 1 in eggs. Dev. Biol. *320*, 402–413.

Iwano, S., Satou, A., Matsumura, S., Sugiyama, N., Ishihama, Y., and Toyoshima, F. (2015). PCTK1 regulates integrin-dependent spindle orientation via protein kinase A regulatory subunit KAP0 and myosin X. Mol. Cell. Biol. *35*, 1197–1208.

Kanda, T., Sullivan, K.F., and Wahl, G.M. (1998). Histone-GFP fusion protein enables sensitive analysis of chromosome dynamics in living mammalian cells. Curr. Biol. *8*, 377–385.

Keith, C.H., Ratan, R., Maxfield, F.R., Bajer, A., and Shelanski, M.L. (1985). Local cytoplasmic calcium gradients in living mitotic cells. Nature *316*, 848–850.

Kittler, R., Putz, G., Pelletier, L., Poser, I., Heninger, A.K., Drechsel, D., Fischer, S., Konstantinova, I., Habermann, B., Grabner, H., et al. (2004). An endoribonuclease-prepared siRNA screen in human cells identifies genes essential for cell division. Nature *432*, 1036–1040.

Lagos-Cabré, R., and Moreno, R.D. (2008). Mitotic, but not meiotic, oriented cell divisions in rat spermatogenesis. Reproduction *135*, 471–478.

Mataragka, S., and Taylor, C.W. (2018). All three IP_3 receptor subtypes generate Ca^{2+} puffs, the universal building blocks of IP_3-evoked Ca^{2+} signals. J. Cell Sci. *131*, jcs220848.

Matov, A., Applegate, K., Kumar, P., Thoma, C., Krek, W., Danuser, G., and Wittmann, T. (2010). Analysis of microtubule dynamic instability using a plus-end growth marker. Nat. Methods *7*, 761–768.

Mitsuyama, F., and Sawai, T. (2001). The redistribution of Ca^{2+} stores with inositol 1,4,5-trisphosphate receptor to the cleavage furrow in a microtubule-dependent manner. Int. J. Dev. Biol. *45*, 861–868.

Nakajima, Y., Meyer, E.J., Kroesen, A., McKinney, S.A., and Gibson, M.C. (2013). Epithelial junctions maintain tissue architecture by directing planar spindle orientation. Nature *500*, 359–362.

Pantazaka, E., and Taylor, C.W. (2011). Differential distribution, clustering, and lateral diffusion of subtypes of the inositol 1,4,5-trisphosphate receptor. J. Biol. Chem. *286*, 23378–23387.

Parry, H., McDougall, A., and Whitaker, M. (2005). Microdomains bounded by endoplasmic reticulum segregate cell cycle calcium transients in syncytial Drosophila embryos. J. Cell Biol. *171*, 47–59.

Pelletier, L., and Yamashita, Y.M. (2012). Centrosome asymmetry and inheritance during animal development. Curr. Opin. Cell Biol. *24*, 541–546.

Poenie, M., Alderton, J., Tsien, R.Y., and Steinhardt, R.A. (1985). Changes of free calcium levels with stages of the cell division cycle. Nature *315*, 147–149.

Poenie, M., Alderton, J., Steinhardt, R., and Tsien, R. (1986). Calcium rises abruptly and briefly throughout the cell at the onset of anaphase. Science *233*, 886–889.

Prole, D.L., and Taylor, C.W. (2016). Inositol 1,4,5-trisphosphate receptors and their protein partners as signalling hubs. J. Physiol. *594*, 2849–2866.

Rossi, A.M., and Taylor, C.W. (2018). IP_3 receptors - lessons from analyses *ex cellula*. J. Cell Sci. *132*, jcs222463.

Sathanawongs, A., Fujiwara, K., Kato, T., Hirose, M., Kamoshita, M., Wojcikiewicz, R.J., Parys, J.B., Ito, J., and Kashiwazaki, N. (2015). The effect of M-phase stage-dependent kinase inhibitors on inositol 1,4,5-trisphosphate receptor 1 (IP_3 R1) expression and localization in pig oocytes. Anim. Sci. J. *86*, 138–147.

Seldin, L., Muroyama, A., and Lechler, T. (2016). NuMA-microtubule interactions are critical for spindle orientation and the morphogenesis of diverse epidermal structures. eLife *5*, e12504.

Shaw, G., Morse, S., Ararat, M., and Graham, F.L. (2002). Preferential transformation of human neuronal cells by human adenoviruses and the origin of HEK 293 cells. FASEB J. *16*, 869–871.

Smyth, J.T., and Putney, J.W. (2012). Regulation of store-operated calcium entry during cell division. Biochem. Soc. Trans. *40*, 119–123.

Smyth, J.T., Petranka, J.G., Boyles, R.R., DeHaven, W.I., Fukushima, M., Johnson, K.L., Williams, J.G., and Putney, J.W., Jr. (2009). Phosphorylation of STIM1 underlies suppression of store-operated calcium entry during mitosis. Nat. Cell Biol. *11*, 1465–1472.

Sugawara, H., Kurosaki, M., Takata, M., and Kurosaki, T. (1997). Genetic evidence for involvement of type 1, type 2 and type 3 inositol 1,4,5-trisphosphate receptors in signal transduction through the B-cell antigen receptor. EMBO J. *16*, 3078–3088.

Thillaiappan, N.B., Chavda, A.P., Tovey, S.C., Prole, D.L., and Taylor, C.W. (2017). Ca^{2+} signals initiate at immobile IP_3 receptors adjacent to ER-plasma membrane junctions. Nat. Commun. *8*, 1505.

Tovey, S.C., Dedos, S.G., Rahman, T., Taylor, E.J.A., Pantazaka, E., and Taylor, C.W. (2010). Regulation of inositol 1,4,5-trisphosphate receptors by cAMP independent of cAMP-dependent protein kinase. J. Biol. Chem. *285*, 12979–12989.

Toyoshima, F., and Nishida, E. (2007). Integrin-mediated adhesion orients the spindle parallel to the substratum in an EB1- and myosin X-dependent manner. EMBO J. *26*, 1487–1498.

Toyoshima, F., Matsumura, S., Morimoto, H., Mitsushima, M., and Nishida, E. (2007). PtdIns(3,4,5)P_3 regulates spindle orientation in adherent cells. Dev. Cell *13*, 796–811.

Whitaker, M., and Patel, R. (1990). Calcium and cell cycle control. Development *108*, 525–542.

Wong, R., Hadjiyanni, I., Wei, H.C., Polevoy, G., McBride, R., Sem, K.P., and Brill, J.A. (2005). PIP_2 hydrolysis and calcium release are required for cytokinesis in *Drosophila* spermatocytes. Curr. Biol. *15*, 1401–1406.

CellPress
OPEN ACCESS

Woźniak, M.J., Bola, B., Brownhill, K., Yang, Y.C., Levakova, V., and Allan, V.J. (2009). Role of kinesin-1 and cytoplasmic dynein in endoplasmic reticulum movement in VERO cells. J. Cell Sci. *122*, 1979–1989.

Yamashita, Y.M. (2009). The centrosome and asymmetric cell division. Prion *3*, 84–88.

Yu, F., Hubrack, S.Z., Chakraborty, S., Sun, L., Alcantara-Adap, E., Kulkarni, R., Billing, A.M., Graumann, J., Taylor, C.W., and Machaca, K. (2019). Remodeling of ER-plasma membrane contact sites but not STIM1 phosphorylation inhibits Ca^{2+} influx in mitosis. Proc. Natl. Acad. Sci. USA *116*, 10392–10401.

Zhao, H., Li, T., Wang, K., Zhao, F., Chen, J., Xu, G., Zhao, J., Li, T., Chen, L., Li, L., et al. (2019). AMPK-mediated activation of MCU stimulates mitochondrial Ca^{2+} entry to promote mitotic progression. Nat. Cell Biol. *21*, 476–486.

STAR★METHODS

KEY RESOURCES TABLE

REAGENT or RESOURCE	SOURCE	IDENTIFIER
Antibodies		
WB, western blot; IC, immunocytochemistry		
Mouse monoclonal anti-α-tubulin (IC, 1:1000)	Sigma Aldrich, Gillingham, UK	cat# T6074; RRID: AB_477582
Rabbit anti-γ-tubulin (IC, 1:400)	GeneTex, CA, USA	cat# GTX113286; RRID: AB_1952442
Rabbit anti-cenexin (aka ODF2) (IC, 1:200-400)	GeneTex	cat# GTX114594; RRID: AB_2814951
Mouse monoclonal anti-β-actin (WB, 1:5000)	Cell Signaling Technology, Danvers, MA, USA	cat# 3700; RRID: AB_2242334
Mouse monoclonal anti-IP_3R1 (WB, 1:1000)	Abclonal, Tokyo, Japan.	cat# A7905; RRID: AB_2770019
Rabbit anti-IP_3R2 (WB, 1:1000)	Pocono Rabbit Farm & Laboratory, Inc., Canadensis, PA, USA	Custom-made for our laboratory (Mataragka and Taylor, 2018)
Mouse monoclonal anti-IP_3R3 (WB, 1:1000; IC, 1:200)	BD Bioscience, NJ, USA	cat# 610313; RRID: AB_397705
Rabbit anti-IP_3R (recognizes all IP_3R subtypes) (WB, 1:1000; Figure 1D).	Pocono Rabbit Farm & Laboratory, Inc.	Custom-made for our laboratory (Mataragka and Taylor, 2018)
Rabbit anti-IP_3R (recognizes all subtypes) (WB, 1:1000; Figure S2D). Although described as anti-IP_3R1, the antigenic peptide is common to all three IP_3R subtypes.	Cell Signaling Technology	cat# 8568; RRID: AB_10890699
Rat monoclonal anti-GFP (WB, 1:2000)	Chromotek, Munich, Germany	cat# 3h9-100; RRID: AB_10773374
Rat monoclonal anti-EB3 antibody (IC, 1:100)	AbCam, Cambridge UK.	cat# Ab53360; RRID AB_880026
IgG-HRP (WB, 1:5000)	Santa Cruz Biotechnology	cat# sc-516102; RRID: AB_2687626
Mouse anti-rabbit IgG-HRP (WB 1:5000)	Santa Cruz Biotechnology	cat# sc-2357; RRID: AB_628497
Goat anti-rat IgG-HRP (WB, 1:5000)	Santa Cruz Biotechnology	cat# sc-2006; RRID: AB_1125219
Alexa Fluor 647 goat anti-rabbit IgG (IC, 1:400)	ThermoFisher	cat# A21244; RRID: AB_141663
Alexa Fluor 488 goat anti-mouse IgG (IC, 1:400)	ThermoFisher	cat# A10667; RRID: AB_2534057
Alexa Fluor 568 goat anti-mouse IgG (IC, 1:400)	ThermoFisher	cat# A11031; RRID: AB_144696
Alexa Fluor 488 goat anti-rat IgG (IC, 1:100)	Thermofisher	cat# A11006; RRID: AB_141373
Alexa Fluor 568 goat anti-rat IgG (IC, 1:100)	Thermofisher	cat# A11077; RRID: AB_141874
Goat anti-rabbit IgG conjugated to CF405M (IC, 1:100)	Sigma	cat# SAB4600461
GFP-Booster ATTO488 nanobody (IC, 1:500)	Chromotek	cat# gba488-100; RRID: AB_2631386
Chemicals, Peptides, and Recombinant Proteins		
Bovine serum albumin (BSA)	Europa Bio-Products, Ely, UK	cat# EQBAH64
Calbryte 590 AM	AAT Bioquest, Sunnyvale, CA, USA	cat# 20700
Carbamoylcholine chloride (carbachol, CCh)	Sigma Aldrich	cat# C-4382
cOmplete, EDTA-free protease inhibitor cocktail	Roche	cat# 11836153001
4′,6-Diamidino-2-phenylindole dihydrochloride (DAPI)	Sigma Aldrich	cat# D9542
3,3′-Dihexyloxacarbocyanine iodide (DHCC)	Sigma Aldrich	cat# 318426
Dulbecco's Modified Eagle's Medium (DMEM)/F-12 with GlutaMAX	ThermoFisher	cat# 31331093
ECL Prime western blotting detection reagent	Amersham	cat# RPN2236
Fibronectin (human)	Merck Millipore, Watford, UK	cat# FC010
Fetal bovine serum (FBS)	Sigma Aldrich	cat# 094N3341
GIBCO TrypLE Express	ThermoFisher	cat# 12605010
HEPES	Merck Millipore	cat# 391338

(*Continued on next page*)

Continued

REAGENT or RESOURCE	SOURCE	IDENTIFIER
HiPerFect	QIAGEN, Hilden, Germany	cat# 301705
Ionomycin	Cambridge Bioscience	cat# CAYM11932
Iscove’s Modified Dulbecco’s Medium (IMDM)	ThermoFisher	cat# 12440-053
NucBlue Live ReadyProbes reagent	ThermoFisher	cat# R37606
PVDF iBlot transfer stack	ThermoFisher	cat# IB401031
Run Blue (4-12%) SDS gel	Expedeon, San Diego, CA, USA	cat# NXG40812
Thymidine	Sigma Aldrich	cat# T9250
TransIT-LT1 reagent	MirusBio, WI, USA	cat# MIR 2300
Tris	ThermoFisher	cat# BP152-1
Tween-20	Sigma-Aldrich	cat# T5927
Trypan Blue	ThermoFisher	cat# T10282
Vectashield	VectorLabs, Burlingame, CA, USA	cat# H-1000
Wheat germ agglutinin (WGA)-CF405M	Biotium, CA, USA	cat# 29028-1
Wheat germ agglutinin (WGA)-CF568	Biotium	cat# 29077-1
Critical Commercial Assays		
QuickChange Lightning site-directed mutagenesis kit	Agilent, Santa Clara, CA USA.	cat# 210515
Experimental Models: Cell Lines		
HEK cells	Dr D. Yule (University of Rochester, NY, USA)	Dr D. Yule (University of Rochester, NY, USA)
HEK-IP$_3$R-KO cells	Kerafast, Boston, MA, USA	cat# EUR030
HEK-IP$_3$R1 cells	Kerafast	cat# EUR031
HEK-IP$_3$R2 cells	Kerafast	cat# EUR032
HEK-IP$_3$R3 cells	Kerafast	cat# EUR033
HAP1 cells	Horizon Discovery, Cambridge, UK	cat# C631
HAP1-IP$_3$R-KO cells	(Atakpa et al., 2018)	Available from Horizon Discovery
Oligonucleotides		
IP$_3$R1 siRNA	QIAGEN	cat# Hs_ITPR1_4 FlexiTube siRNA (SI00034545)
IP$_3$R2 siRNA	QIAGEN	cat# Hs_ITPR2_1 FlexiTube siRNA (SI00034552)
IP$_3$R3 siRNA	QIAGEN	cat# Hs_ITPR3_1 FlexiTube siRNA (SI00034580)
Non-silencing (NS) siRNA	QIAGEN	cat# 1027281
Recombinant DNA		
pcDNA3.2/DEST-EGFP-IP$_3$R3 (rat)	(Pantazaka and Taylor, 2011)	N/A
pcDNA3.2/V5DEST-IP$_3$R3 (rat)	(Tovey et al., 2010)	N/A
pShuttle mCherry-α-tubulin	Addgene	Addgene plasmid # 26768; RRID: Addgene_26768 (Matov et al., 2010)
Histone 2B-EGFP	Addgene	Addgene plasmid # 11680; RRID: Addgene_11680 (Kanda et al., 1998)
GFP-ER		(Woźniak et al., 2009)
Software and Algorithms		
GraphPad Prism 6.0	GraphPad Software, 6.0	https://www.graphpad.com/
Excel	Microsoft, 2007	N/A
FIJI		https://fiji.sc/
GeneTools, version 4	Syngene, Cambridge, UK	https://www.syngene.com/
MetaMorph Microscopy Automation and Image Analysis	Molecular Devices, San Jose, CA	https://www.moleculardevices.com

RESOURCE AVAILABILITY

Lead Contact

Further information and requests for resources and reagents should be directed to, and will be fulfilled by, the Lead Contact, Colin W Taylor (cwt1000@cam.ac.uk).

Materials Availability

This study did not generate any unique materials. All materials needed to support the claims of the study are available commercially.

Data and Code Availability

This study did not generate any unique dataset or code that is required to support the claims of the paper.

EXPERIMENTAL MODEL AND SUBJECT DETAILS

We used two cell lines, human embryonic kidney (HEK) 293 cells and HAP1 cells (near-haploid) and their derivatives. At the time of the study, these were the only mammalian cell lines in which all three IP_3R subtypes had been disrupted. HEK293 cells are hypotriploid cells derived from embryonic kidney, immortalized by transformation with adenovirus, and with features suggesting derivation from a neural lineage (Shaw et al., 2002). The four HEK cell lines used (HEK-IP_3R-KO or expressing only one of the three IP_3R subtypes) were generated in Dr D. Yule's laboratory using CRISPR/Cas9 (Alzayady et al., 2016). The edited cell lines were supplied by Kerafast (Boston, MA, USA), and the parental cell line (WT), from which the edited cells were generated, was provided by Dr Yule (University of Rochester, NY, USA). HAP1 cells are human, near-haploid, fibroblast-like cells derived from the chronic myelogenous leukemia (CML) cell line, KBM-7. The cell line in which genes encoding all three IP_3R subtypes were disrupted by CRISPR/Cas9 (HAP1 IP_3R-KO) has been described previously (Atakpa et al., 2018). We have not independently verified the authenticity of the cell lines.

METHOD DETAILS

Cell Culture and Transfection

HEK cells were cultured in DMEM/F-12 with GlutaMAX and fetal bovine serum (FBS, 10%) at 37°C in humidified air with 5% CO_2. HAP1 cells were cultured in IMDM with FBS (10%) at 37°C in humidified air with 5% CO_2. Cells were passaged every 3-4 days using GIBCO TrypLE Express. Regular screening confirmed that all cells were free of mycoplasma.

For imaging, cells were grown on 16-mm round glass coverslips (N° 0, VWR, International, Radnor, PA, USA) or 35-mm glass-bottomed imaging dishes (#P35G-1.0-14-C, MatTek Corporation, Ashland, MA, USA) coated with human fibronectin (50 μg/mL).

Transient transfection with pcDNA3.2/V5DEST-IP_3R3, pcDNA3.2/DEST-EGFP-IP_3R3, pcDNA3.2/DEST-EGFP-$IP_3R3^{D/A}$, pShuttle mCherry-α-tubulin or H2B-EGFP plasmids used TransIT-LT1 reagent (1 μg DNA/3 μL reagent) according to the manufacturer's instructions. Transfection with siRNA against each IP_3R subtype (40 nM of each) or a non-silencing (NS) siRNA (120 nM) used HiPerfect transfection reagent according to the manufacturer's instructions. Cells were used after 48 hr. pcDNA3.2/DEST-EGFP-IP_3R3 was used to modify the coding sequence of EGFP-IP_3R3 from D2477 (CGA) to A (GCC) using primers 5′-TGCGGAGGATGGCGCC CACGCCG-3′ and 5′-CGGCGTGGGCGCCATCCTCCGCA-3′ and the QuickChange Lightning site-directed mutagenesis kit according to the manufacturer's instruction. Sequencing of the entire coding sequence confirmed the single mutation.

Most experiments used non-synchronized cells, but to obtain sufficient cells in a field for analyses of isolated cells, cells were synchronized using either a single- or double-thymidine block. Thymidine arrests cells at the G1/S boundary by inhibiting DNA synthesis, and cells then synchronously enter S phase when thymidine is removed. For the single-block, HEK cells (10^5/well) grown on 35-mm imaging dishes coated with human fibronectin (50 μg/mL) were incubated with thymidine (2 mM) for 18-24 hr, washed three times in phosphate-buffered saline (PBS) and incubated in fresh medium for 7-10 hr before analysis. For the double-block, cells were incubated with thymidine (2 mM, 18 hr), washed, incubated in thymidine-free medium (9 hr), incubated again with thymidine (2 mM, 18 hr), washed and incubated in fresh medium (8-10 hr). We used these methods to avoid synchronization protocols that rely on perturbation of microtubules.

A Countess automated cell counter (ThermoFisher) was used to count cells in medium containing 0.2% Trypan Blue.

Immunocytochemistry

Cells (10^5/well) grown (24 hr) on 16-mm coverslips were fixed in ice-cold methanol, permeabilized in Tris-buffered saline (TBS: 50 mM Tris-HCl, 150 mM NaCl, pH 7.5) with Tween-20 (0.1%) and Triton X-100 (0.1%), and then blocked in TBS containing Tween (0.1%) and BSA (2%). Cells were incubated with primary antibody (1 hr, 20°C), washed three times with TBS containing 0.1% Tween, and incubated with fluorescent secondary antibodies (1 hr, 20°C). DAPI (300 nM, 10 min, 20°C) was used to stain nuclei. Fluorescently tagged WGA (5 μM, 10 min 37°C, added before fixing cells) was used to identify the plasma membrane. Coverslips were mounted with Vectashield on microscope slides over two stripes of nail polish, sealed with nail polish, and observed using an Olympus IX83 microscope with 60x and 100x objectives (numerical apertures, NA, 1.3 and 1.49 respectively). For total internal reflection fluorescence microscopy (TIRFM) the penetration depth was 90-140 nm. An iLas2 illumination system (Cairn, Faversham, Kent, UK) was

used for TIRFM and wide-field imaging. For quantification of IP_3R3 immunostaining (Figures S3A and S3B), we used the background-corrected complete z stack from confocal images.

Live-Cell Imaging

Cells transfected to express mCherry-α-tubulin alone or with H2B-EGFP, and grown in imaging dishes were washed and incubated in HEPES-buffered saline (HBS: 135 mM NaCl, 5.9 mM KCl, 1.2 mM $MgCl_2$, 1.5 mM $CaCl_2$, 11.5 mM glucose, 11.6 mM HEPES, pH 7.4) containing FBS (10%). Wide-field images of cells in HBS were collected at 1-min intervals using an Olympus IX83 microscope with a 100x objective (NA, 1.49) within an enclosed cabinet (37°C, 5% CO_2) using 488/525 nm (excitation laser/emission filter) and 561/630 nm lasers, and an iLas2 targeted laser illumination system.

Measurement of Contact Areas Between Cells and Substratum

Synchronized HEK cells were incubated in HBS with 3,3′-dihexyloxacarbocyanionie iodide (DHCC, 20 μg/mL, ~1 min). Live cells were imaged within 20 min by TIRFM and confocal microscopy (488/525 nm) using an Olympus IX83 microscope with 100x objective (NA, 1.49) to determine the areas contacting the substratum and across a mid-section of each cell.

Measurements of Cytosolic Ca^{2+} Signals

HEK cells in HBS were incubated with Calbryte 590-AM (5 μM, 1 hr, 20°C in darkness), washed and incubated in HBS (45 min, 20°C) before imaging. Wide-field fluorescence images (488/525 nm to detect EGFP, 561/630 nm to detect Calbryte 590) were collected at 500-ms intervals using an Olympus IX83 microscope with a 100x objective (NA, 1.49). After subtraction of background fluorescence (from an area outside the cell), changes in fluorescence (F/F_0) are reported relative to basal fluorescence (F_0).

Western Blots

Cells were scraped into cold RIPA medium (1 mM Tris HCl, 15 mM NaCl, 0.5 mM EDTA, 0.1% Triton X-100, pH 7.5) containing protease inhibitors (cOmplete, EDTA-free protease inhibitor cocktail) and lysed using a syringe and 18G needle. The protein content of the supernatant (10,000 x*g*, 10 min) was quantified using a Bradford assay with BSA as standard. Proteins were separated using 4%–12% Run Blue Bis-tris gels, transferred to a polyvinyl difluoride (PVDF) membrane using an iBlot gel-transfer system, blocked (1 hr, 20°C) in TBS with Tween-20 (0.1%) and BSA (5%), and incubated (1 hr, 20°C) with primary antibody in fresh blocking buffer. After three washes, the membrane was incubated with HRP-conjugated secondary antibody (1 hr, 20°C) and washed three times. Bands were visualized with ECL Prime western blotting detection reagent using a GeneTools Syngene PXi chemiluminescence detection system, and quantified using FIJI (after subtracting a background measured from an area adjacent to the relevant band). Band intensities were expressed relative to control bands on the same gel.

QUANTIFICATION AND STATISTICAL ANALYSIS

Measurements of Angles of Mitotic Spindles and NuMA Crescents

We used a published method to measure spindle angle relative to the substratum (Toyoshima et al., 2007; Toyoshima and Nishida, 2007). Briefly, confocal z stack images (0.5-μm thick) of samples stained for α- or γ-tubulin, or transfected with mCherry-α-tubulin, were projected as a 3D image to obtain a side view using FIJI software 3D project plugin. The distance between the mitotic poles (A) and the height difference between them (Z) was measured (spindle angle = ArcSin(Z/A)) (Figure 1A).

For NuMA crescent angles, the difference between the centers of each crescent in a z stack (Z_1-Z_2) and their separation in the x-plane (X) were measured (NuMA angle = ArcTan((Z1-Z2)/X)) (Figure S4E).

Measurements of IP_3R Distributions Around Mother and Daughter Centrosomes

Mother and daughter centrosomes were distinguished by immunostaining for cenexin, which more intensely labels the mother centrosome (Colicino et al., 2019). The confocal section in which each centrosome was most intensely labeled was then used to analyze IP_3R distribution (immunostained or EGFP) around that centrosome. For each section, the average fluorescence intensity (mean ± SD) was determined, and pixels surrounding each centrosome that exceeded a threshold (mean + 2SD) were used to calculate areas occupied by IP_3Rs (Figure 3B).

Measurements of Lengths of Astral Microtubules

We used two different methods to measure the length of astral microtubules: tracing anti-α-tubulin staining from each centrosome (Figures 3C and 3D) and measuring distances from centrosomes to immunostained EB3 (Figures S4C–S4G). Cells immmunostained for cenexin, α-tubulin and/or EB3 and/or with the plasma membrane identified by WGA-staining were imaged using spinning-disk confocal microscopy. After background correction, Z stack projections of five sections that included each centrosome were used to measure the lengths of astral microtubules in the area generated by projecting a line passing through each centrosome, perpendicular to the spindle axis using FIJI (Figure S4E). The same methods were used to quantify cortical EB3 by counting immunostained EB3 puncta associated with WGA.

Statistical Analyses

Statistical analyses used GraphPad Prism version 6. Results are presented as means ± SD or SEM, as appropriate. Paired or unpaired Student's t tests (for 2 variables) or one-way ANOVA with Bonferroni post hoc test (for multiple comparisons) was used for statistical analyses (* $p < 0.05$, ** $p < 0.01$ and *** $p < 0.001$). χ^2 test for trend was used for comparisons of frequency distributions. Sample sizes and the tests used are provided in figure legends.

Cell Reports, Volume *33*

Supplemental Information

Ca^{2+} Release by IP_3 Receptors Is Required to Orient the Mitotic Spindle

Raul Lagos-Cabré, Adelina Ivanova, and Colin W. Taylor

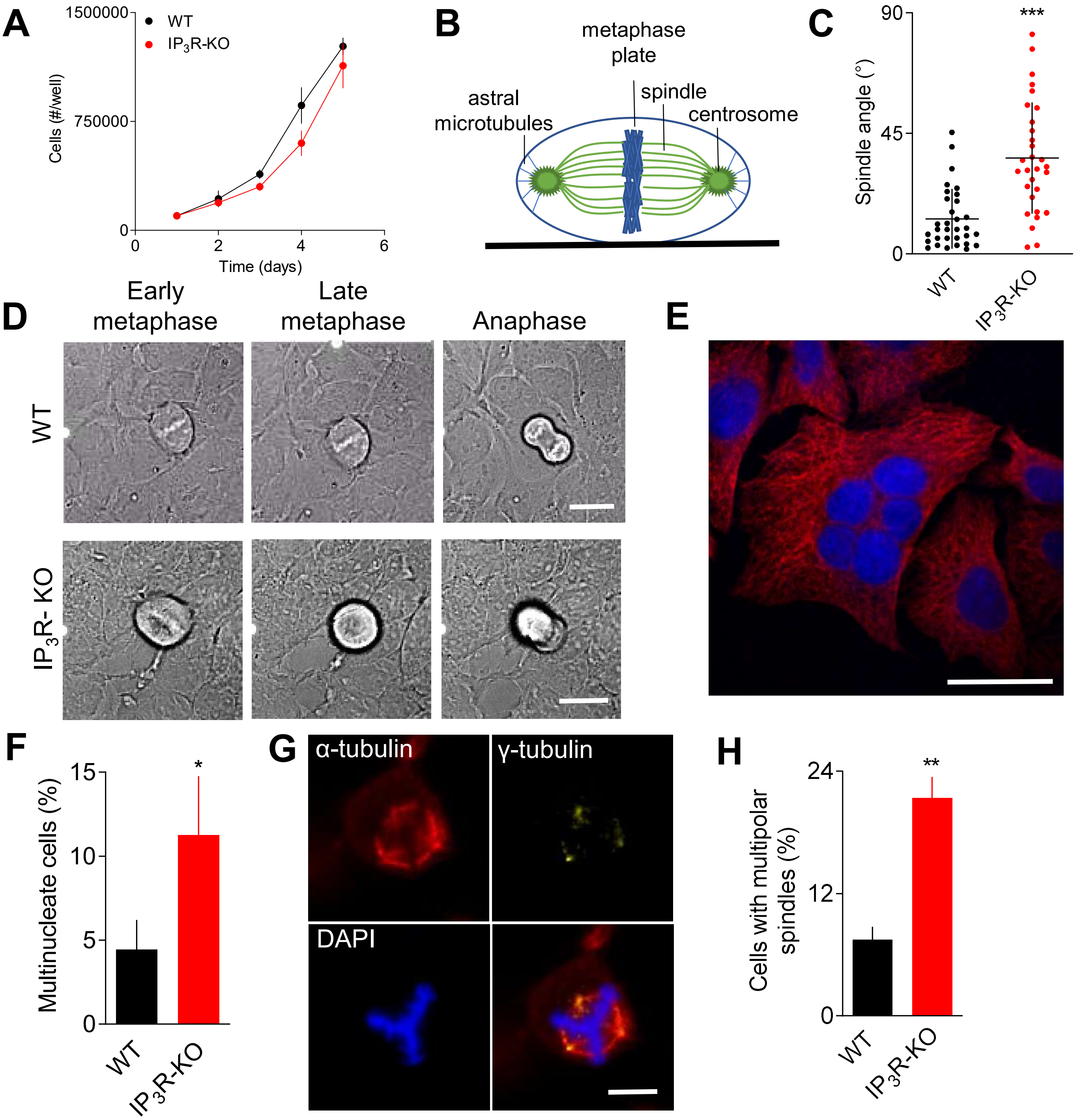

Figure S1. Cells Without IP_3Rs Proliferate Normally But With Aberrant Spindles. Related to Figure 1.

(A) Numbers of cells/well for cells plated on day 1 with 100,000 cells/well. Mean ± SD from 3 experiments.

(B) In normal cells, the metaphase plate aligns perpendicular to the substratum, with the mitotic spindle parallel to it.

(C) Spindle angles measured in fixed WT and HEK-IP_3R-KO cells using γ-tubulin staining to identify centrosomes. Individual values mean ± SD from 3 experiments. $^{***}P < 0.001$, Student's *t*-test.

(D) Brightfield images show that both daughter cells remain attached to the substratum during anaphase of a WT cell, while in the HEK-IP_3R-KO cell only one daughter cell remains attached. Scale bars = 20 µm

(E) Image of a multinucleate HEK-IP_3R-KO cell stained with α-tubulin (red) and DAPI (blue). Scale bar = 20 µm.

(F) Summary shows numbers of multinucleate cells. Mean ± SEM, $n = 8$ experiments, with 10 cells analysed in each. $^{*}P < 0.05$, Student's *t*-test.

(G) Typical example of a multipolar spindle in a HEK-IP_3R-KO cell stained for α-tubulin (red), γ-tubulin (yellow) and DNA (blue). Scale bar = 10 µm.

(H) Summary shows numbers of cells with multipolar spindles. Mean ± SEM, $n = 3$ dishes with 11 fields quantified in each. $^{**}P < 0.01$, Student's *t*-test.

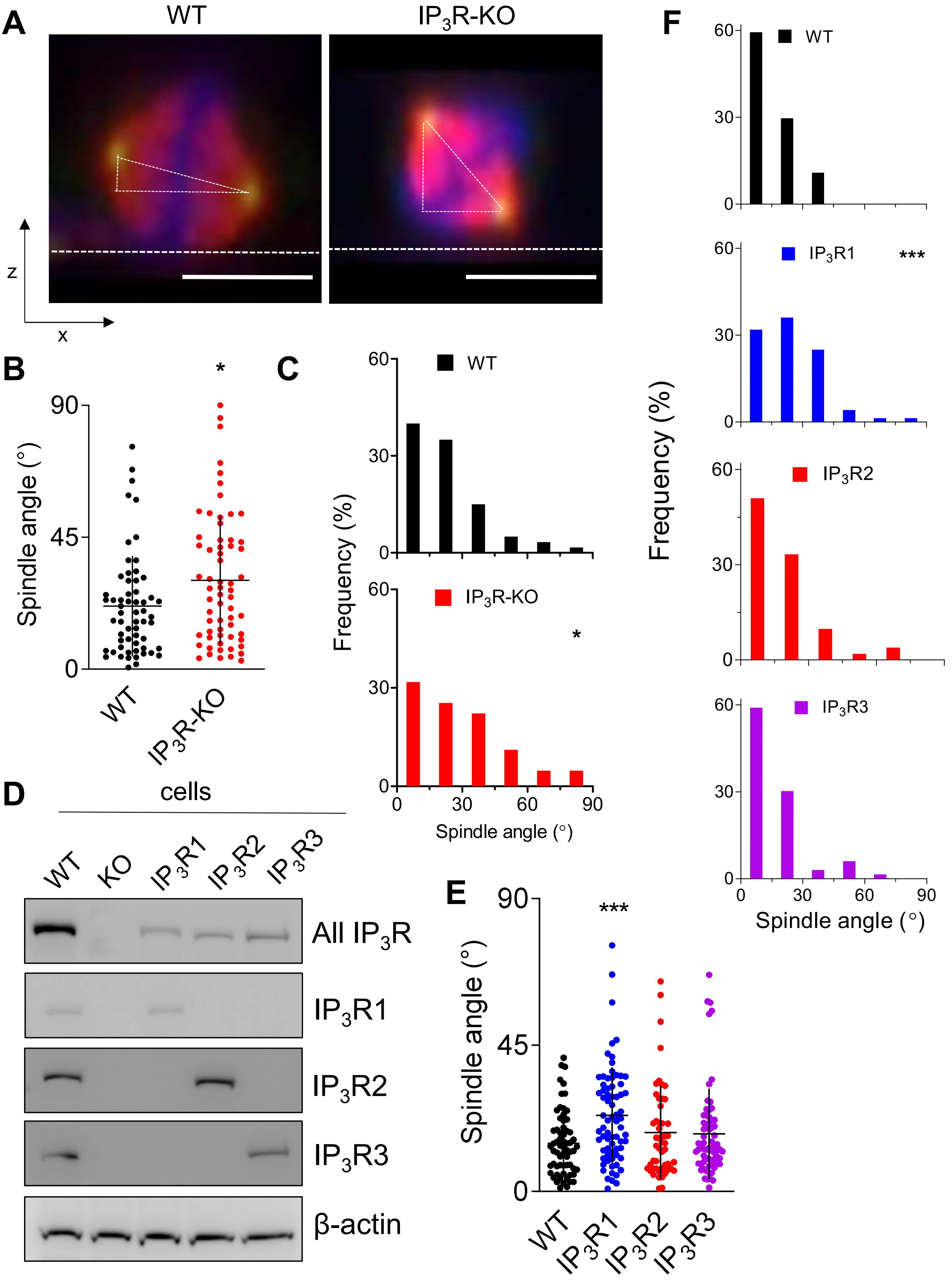

Figure S2. Spindle Angles in HAP1 Cells Without IP_3Rs and in HEK Cells Expressing Single IP_3R Subtypes. Related to Figure 1.
Legend on next page.

Figure S2. Spindle Angles in HAP1 Cells Without IP_3Rs and in HEK Cells Expressing Single IP_3R Subtypes. Related to Figure 1.
Figure on preceding page.

(A) Confocal z-stack images from fixed HAP1 cells show spindle angles during metaphase for a WT and HAP1-IP_3R-KO cell. The cells are immunostained for α-tubulin (red) and γ-tubulin (green), and stained with DAPI (blue) for chromosomes. Scale bars = 10 μm.
(B) Spindle angles for WT and HAP1-IP_3R-KO cells. Individual values, mean ± SD from 5 experiments. $^{*}P < 0.05$, Student's *t*-test.
(C) Frequency distribution of the spindle angles (data from panel B). $^{*}P < 0.05$, relative to WT, χ^2 test for trend.
(D) Typical western blots, using antibodies selective for IP_3R subtypes (IP_3R1-3) or an antibody that recognizes all three IP_3R subtypes (all IP_3R), show expression of IP_3R subtypes in WT HEK cells, HEK-IP_3R-KO cells and cells expressing single native IP_3R subtypes (Alzayady et al., 2016). The results replicate our previous analyses, which established that the HEK cell line used expresses 36% IP_3R1, 18% IP_3R2 and 45% IP_3R3 (Mataragka and Taylor, 2018).
(E) Spindle angles of cells expressing single IP_3R subtypes. Results show individual values, mean ± SD from 5 experiments. $^{***}P < 0.001$ relative to WT, ANOVA with Bonferroni test.
(F) Frequency distribution of spindle angles (data from panel B). $^{***}P < 0.001$, relative to WT, χ^2 test for trend.

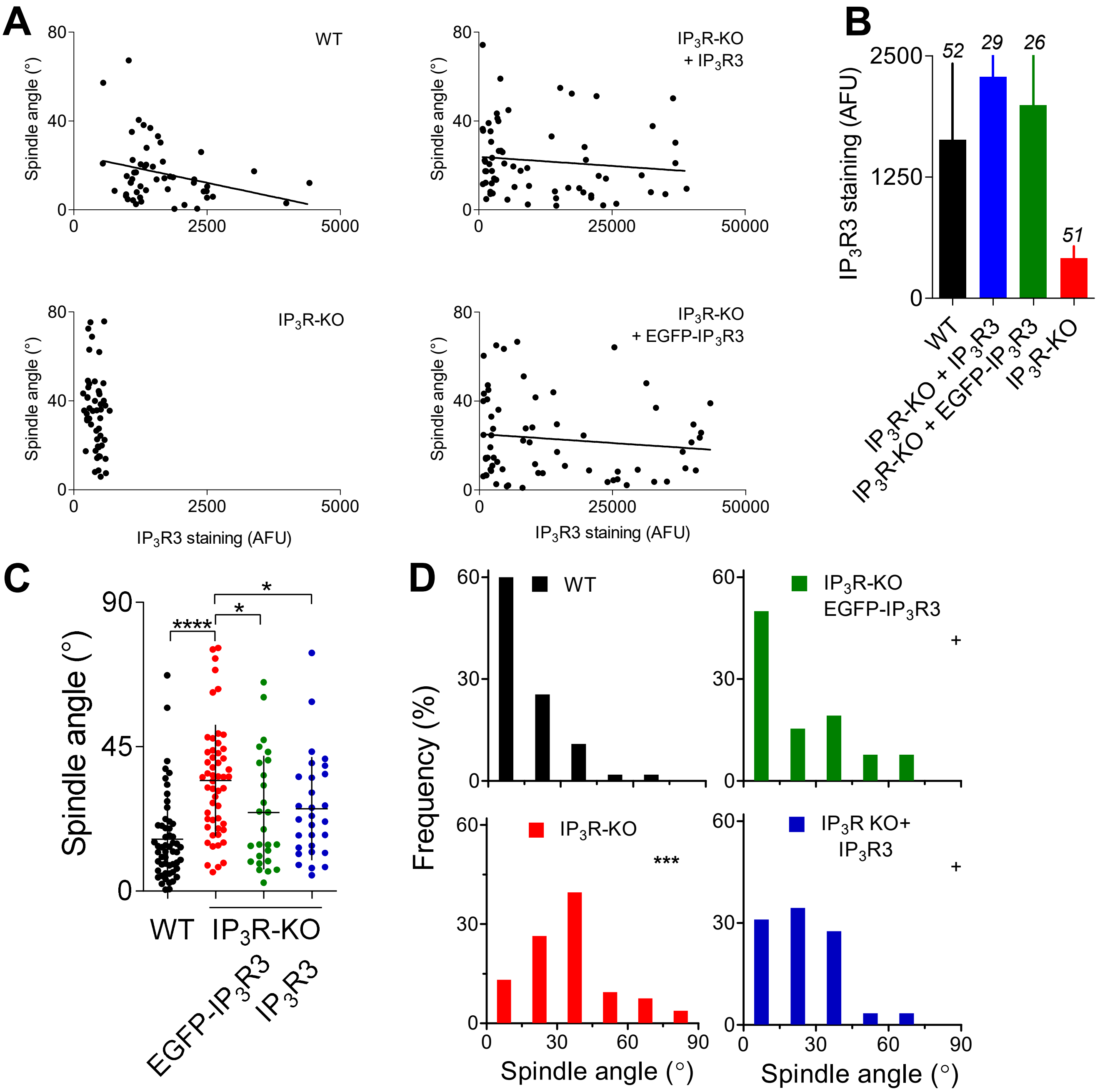

Figure S3. IP_3R3 and EGFP-IP_3R3 Similarly Rescue Spindle Alignment. Related to Figure 1.

(A) Relationships between immunostaining for IP_3R3 and spindle angle measured under identical conditions for WT and HEK-IP_3R-KO cells, or the latter expressing IP_3R3 or EGFP-IP_3R3. Points are from individual cells taken from 5 experiments. AFU, arbitrary fluorescence units (note the ten-fold difference in scale for WT and IP_3R-KO cells relative to transfected cells). For IP_3R-KO cells, all cells contributed to the analysis; whereas for the other cells only those with detectable immunostaining were included. From least-squares linear regression, the only slope to differ significantly from 0 was for WT cells ($P < 0.0001$). The results suggest firstly that the level of IP_3R expression in WT cells may contribute to whether the spindle aligns appropriately, and secondly that even massive over-expression of IP_3R does not perturb spindle alignment. Summary results in **Figures 1I and 1J**.

(B) Cells heterologously expressing IP_3R3 were selected (by immunostaining) to approximately match WT in their expression of IP_3R3. Note that in WT cells IP_3R3 comprises only ~45% of the IP_3Rs expressed (Mataragka and Taylor, 2018), whereas IP_3R3 is the only subtype in heterologously-expressing cells. Results show relative expression levels of IP_3R3 for cells used in the analyses shown in panels C and D.

(C) Spindle angles measured in the indicated cells, approximately matched for their expression of IP_3R3 (see panel B). Individual values, mean ± SD. $^{****}P < 0.0001$, $^{*}P < 0.05$, one-way ANOVA with Holm-Sidak multiple comparisons test, relative to HEK-IP_3R-KO cells.

(D) Distributions of spindle angles (data from panel C). $^{***}P < 0.001$, relative to WT, $^{+}P < 0.05$ relative to IP_3R-KO, χ^2 test for trend. Results shown here comprise a subset of the data (cells with IP_3R3 expression approximately matching expression in WT cells) shown in **Figures 1I and 1J**.

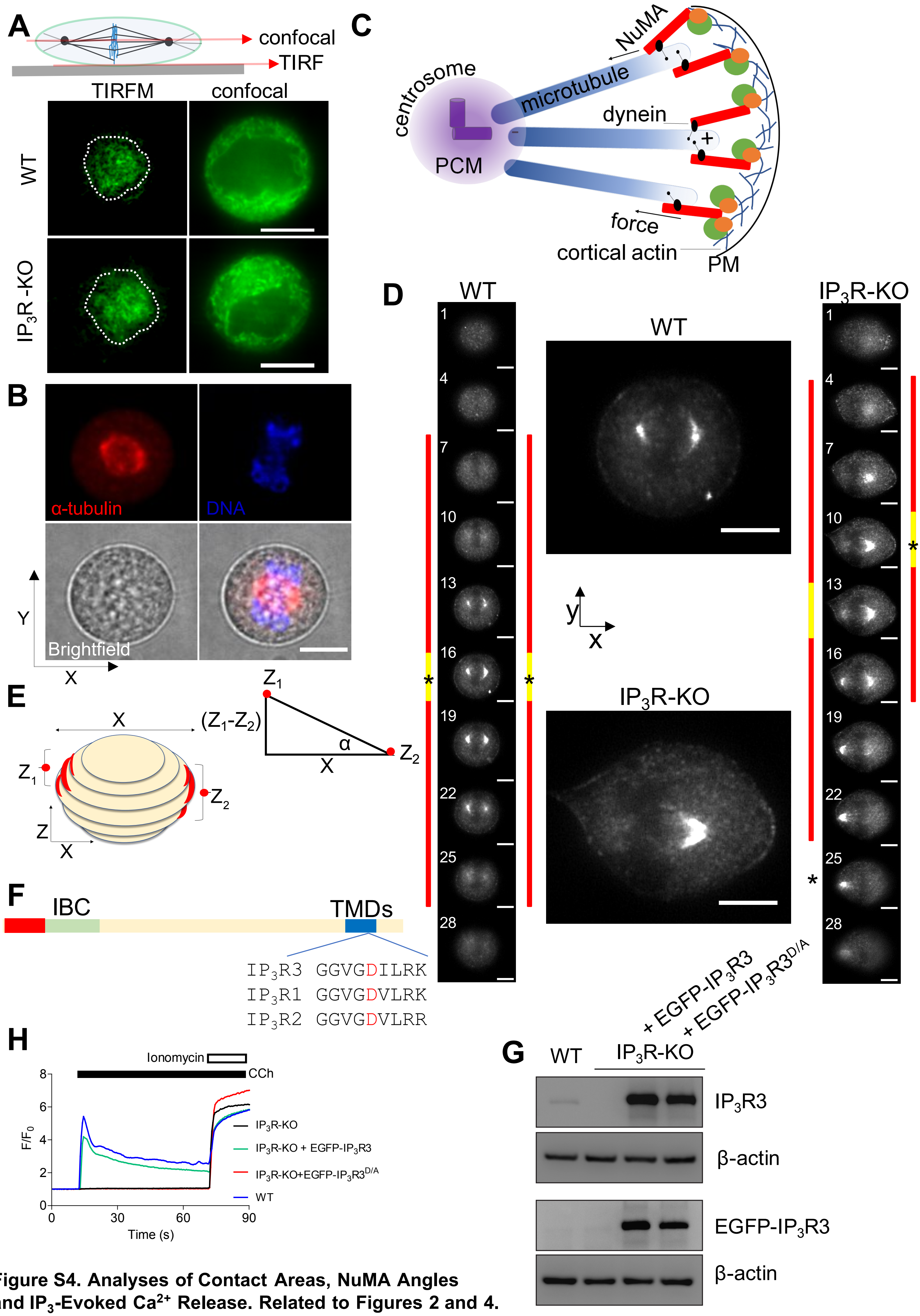

Figure S4. Analyses of Contact Areas, NuMA Angles and IP$_3$-Evoked Ca^{2+} Release. Related to Figures 2 and 4. Legend on next page.

Figure S4. Analyses of Contact Areas, NuMA Angles and IP_3-Evoked Ca^{2+} Release. Related to Figures 2 and 4.
Figure on preceding page.

(A) Confocal and TIRF microscopy was used to measure contact areas with the substratum for dividing cells with membranes labelled using DHCC (green). Scale bars = 10 μm. Summary results in **Figure 2A**.
(B) Typical confocal sections and brightfield images of isolated synchronized WT HEK cell expressing mCherry-α-tubulin and with DNA stained with NucBlue. Scale bar = 10 μm. Similar images were used to provide z-stacks from which spindle angles were measured (**Figures 2B-D**).
(C) NuMA links dynein to microtubules, and, through associated proteins, it associates with cortical actin. PCM, pericentriolar material.
(D) Examples of WT and HEK-IP_3R-KO cells in metaphase immunostained for NuMA. The large images show a single confocal section that includes a centrosome. Small images are sections (numbered from base of cell) showing locations of centrosomes (*) and the bounds of each NuMA crescent (red bars) and its centre (yellow bars, from which NuMA angles were calculated). NuMA angles were measured between the central points of the NuMA crescents relative to the substratum (**Figures 2E and 2F**). Scale bars = 10 μm.
(E) NuMA angles relative to the substratum were calculated as shown. The z-planes in which the centre of each NuMA crescent was located were identified (Z_1 and Z_2) and the angle between them (α) was calculated.
(F) Location of the mutated residue (D2477 of IP_3R3) within the selectivity filter that is conserved within all three IP_3R subtypes and falls within the re-entrant luminal loop linking transmembrane domains (TMD) 5 and 6. IBC, IP_3-binding core.
(G) Typical western blot (20 μg protein/lane) using antibodies to IP_3R3 (top) or GFP (bottom) of WT HEK cells or HEK-IP_3R-KO cells expressing the indicated IP_3R3 proteins. In 4 independent paired analyses, the intensity of the IP_3R3 band (determined using an Ab for IP_3R3) for cells expressing EGFP-$IP_3R3^{D/A}$ was 81.2 ± 4.5% that from cells expressing EGFP-IP_3R3.
(H) Ca^{2+} signals recorded using Calbryte-590 from HEK cells transiently expressing EGFP-IP_3R3s. Carbachol (CCh, 100 μM) was added to stimulate IP_3 formation through endogenous muscarinic acetylcholine receptors. Ionomycin (100 μM) was added to allow saturation of the Ca^{2+}-indicator. Results (F/F_0, where F is fluorescence recorded at each time, and F_0 fluorescence recorded at t = 0) are means from at least 22 successfully transfected cells from 3 experiments. The results establish that $IP_3R3^{D/A}$ does not mediate IP_3-evoked Ca^{2+} release (Boehning et al., 2001; Dellis et al., 2008).

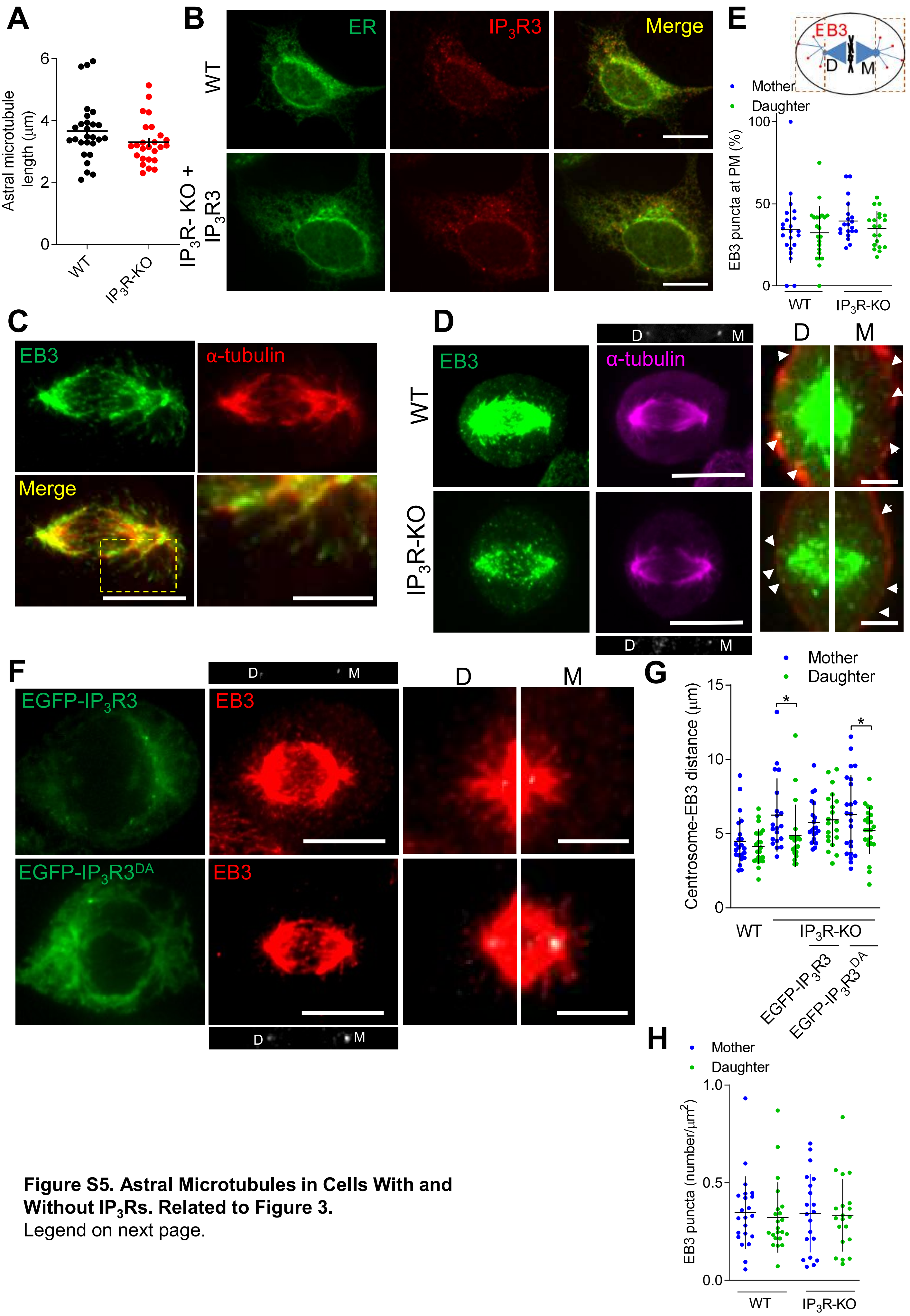

Figure S5. Astral Microtubules in Cells With and Without IP$_3$Rs. Related to Figure 3.
Legend on next page.

Figure S5. Astral Microtubules in Cells With and Without IP_3Rs. Related to Figure 3.
Figure on preceding page.

(A) Z-stacks from confocal sections that included both centrosomes (identified by cenexin staining) (**Figure 3C**) were used to measure lengths of astral microtubules at each pole of each cell (~8 measurements per cell). Results show mean values for each cell (without distinguishing mother and daughter centrosomes), and mean ± SEM for all cells. No significant difference between WT and HEK-IP_3R-KO cells, Student's *t*-test.
(B) Typical confocal images (central plane) show ER (GFP-ER, green) and immunostained IP_3R3 (red) in interphase WT HEK cells and HEK-IP_3R-KO cells expressing IP_3R3. Scale bars = 10 μm. Results confirm the similar subcellular distributions of native and heterologously expressed IP_3R3.
(C) Confocal image of HEK-IP_3R-KO cell immunostained for EB3 (green) and α-tubulin (red), showing EB3 capping astral microtubules. Scale bars = 10 μm (5 μm in enlargement of boxed area).
(D) Maximum intensity z-stack images around centrosome of HEK cells immunostained for EB3 (green), α-tubulin (magenta) and cenexin (grey), and with the plasma membrane (PM) identified using wheat germ agglutinin (WGA, red). Arrows show EB3 at the cell cortex (PM). Scale bars = 10 μm (5 μm in enlargements of areas surrounding centrosomes). D, daughter; M, mother.
(E) Summary results show the fraction of EB3 puncta located at the PM around mother and daughter centrosomes (areas shown in the inset) for cells with (WT, n = 22 cells) or without IP_3Rs (IP_3R-KO, n = 20 cells). Results show individual values and mean ± SD. No significant difference between any values, one-way ANOVA.
(F) Maximum intensity z-stack images around centrosome immunostained for EB3 (red) and cenexin (grey) in HEK-IP_3R-KO cells transfected with EGFP-IP_3R3 or EGFP-IP_3R3^{DA}. GFP-Booster was used to enhance the fluorescence of EGFP. Scale bars = 10 μm (5 μm in enlargements of regions surrounding centrosomes).
(G) Summary results show distance between centrosomes (cenexin) and EB3 in WT cells and HEK IP_3R-KO cells alone or after expression of EGFP-IP_3R3 or EGFP-IP_3R3^{DA}. Results show individual values, mean ± SD. $^*P < 0.05$, Paired student's *t*-test, mother relative to daughter centrosome.
(H) Numbers of immunostained EB3 puncta (#/μm^2) identified in regions around each centrosome (see inset to panel E). Results show individual values with mean ± SD from 22 (WT) or 20 (IP_3R-KO) cells. No significant differences, one-way ANOVA.
